# Sporicidal and bactericidal efficacy of plasma-treated liquids based on reaction kinetics of peroxynitrous acid

**DOI:** 10.3389/fmicb.2026.1788374

**Published:** 2026-03-10

**Authors:** Alexander Pogoda, Veronika Hahn, Klaus-Dieter Weltmann, Juergen F. Kolb

**Affiliations:** 1 Leibniz Institute for Plasma Science and Technology (INP), Greifswald, Germany; 2 Institute of Physics, University of Rostock, Rostock, Germany

**Keywords:** antimicrobial efficacy, bacterial spores, buffer capacity, *Escherichia coli*, peroxynitrous acid, plasma-treated liquids, reaction kinetics, reactive oxygen and nitrogen species

## Abstract

**Introduction:**

Plasma-treated aqueous solutions have proven effective for the inactivation of bacteria and even dormant spores. However, reported efficacies vary considerably across setups and experimental conditions. Consequently, different reactive species formed during treatment and their specific reaction kinetics are considered to be responsible. Their individual contribution to microbial inactivation depends on a thorough understanding of the underlying chemical processes. We found that the buffer capacity of an aqueous solution strongly influences the concentrations of reactive species required for effective microbial inactivation. Conversely, the temporal evolution of reactions allows for the optimization of bactericidal and sporicidal efficacy.

**Methods:**

Using time-resolved *in situ* UV spectrometry, formation and degradation processes of significant reactive oxygen and nitrogen species (RONS) were observed and analyzed during and after plasma treatment.

**Results:**

The availability and concentration of peroxynitrous acid (ONOOH) proved crucial for the antimicrobial activity of the liquid. ONOOH generation depends on hydrogen peroxide (H_2_O_2_) and nitrite (NO_2_^–^), both supplied by the plasma exposure, and eventually decays to nitrate (NO_3_^–^), which remains in solution. Experimental data showed that liquids with higher buffer capacity accumulated higher concentrations of H_2_O_2_ and NO_2_^–^ during plasma exposure, enabling continued ONOOH production even after partial buffer depletion. Concurrently, the solutions acidified progressively. Bacteria, either vegetative cells or dormant spores, were added to the solutions at different time points during the process, and inactivation was monitored in relation to RONS concentrations. The observed antimicrobial efficacy correlated directly with ONOOH concentration, which can be adjusted via the buffer capacity of the medium. This resulted in a 3.83-log_10_ reduction of *Bacillus atrophaeus* spores within 90 min and a 5.78-log_10_ reduction of *Escherichia coli* within 45 min.

**Discussion:**

Simulations reproduced these experimental trends, confirming three distinct kinetic regimes: a pre-reaction window (before ONOOH formation), a main reaction window (dominated by ONOOH production), and a post-reaction window (defined by decomposition).

## 1 Introduction

Cold (non-thermal) plasmas have a proven potential for the inactivation of infectious pathogens. Respective exposures have been successful for the treatment of chronic wounds (Arndt et al., 2018; Boeckmann et al., 2020; Metelmann et al., 2018), the disinfection of surfaces and medical devices (Kong et al., 2009; Weltmann and von Woedtke, 2016), the sanitation of fresh produce and seeds (Misra et al., 2016; Surowsky et al., 2016; Zhao et al., 2020), indoor air hygiene, and the inactivation of waterborne microorganisms (Kramer et al., 2015; Prehn et al., 2020). Decontamination by a non-thermal plasma is based on the generation of reactive species from air and water. Reaction kinetics, especially for plasma-treated liquids are considered to promote a biocidal response. This could be mediated, for example, by wound secretions or by deliberately encouraging an interaction with aqueous solutions. Accordingly, media of different compositions can obtain biocidal characteristics that can be retained for some time. The plasma-treated medium (PTM) can, hence, be used for decontamination. For this “indirect plasma treatment” (which comprises the main part of our study), the medium is exposed to plasma and afterwards the microorganisms can be inactivated with this PTM. On the contrary, during “direct plasma treatment” the microorganisms are already in the solution, which is treated. In both cases, reactive species that are generated in the plasma can either be transported from the gas phase into the liquid or be formed in the gas–liquid interface itself (Lukeš et al., 2012; Shen et al., 2019). The efficacy of plasma treatments (for example for any microbial inactivation) depends on the individual differences of specific approaches and on the subsequent fate of reactive species in the liquid. An early instructive example for the successful use of PTM for disinfection was given by Ikawa et al. (2016). The study described a decontamination process in which water was treated with plasma and then mixed with a bacterial suspension. The study showed that, in particular, peroxynitrous acid was responsible for the antimicrobial effect. Additionally, the first evidence that the treatment of aqueous liquids by a surface dielectric-barrier discharge (DBD) operated in atmospheric air leads to an antimicrobial effect of the liquid itself was provided by Oehmigen et al. (2011). Even 30 min after the plasma was switched off, *Escherichia coli* (*E. coli*) that were added to the treated sodium chloride solution were still inactivated. The authors again hypothesized that the formation of peroxynitrous acid was primarily responsible for this effect and that respective concentrations, which decreased over time eventually, reduced the antimicrobial efficacy.

When cold plasmas are ignited and operated in air, electrons excite air molecules via collision processes and the primarily formed species are atomic nitrogen and oxygen, hydrated (solvated) electrons (e*_*solv*_*), hydroxyl radicals (OH⋅), and hydrogen superoxide (HO_2_) (Bruggeman et al., 2016). These are generally considered rather short-lived. The species react to compounds such as hydrogen peroxide (H_2_O_2_), ozone (O_3_), nitric acid (HNO_3_), or nitrous acid (HNO_2_). Many of these species are known as antimicrobial agents, and although they are generally longer-lived, they will eventually decay in an aqueous environment. Conversely, prominent, relatively stable compounds that persist long after plasma exposure are nitrate ($\text{N}\,{\text{O}}_{3}^{-}$), nitrite ($\text{N}\,{\text{O}}_{2}^{-}$), and H_2_O_2_. The latter can react with nitrite to peroxynitrous acid (ONOOH). The reaction depends on, and is associated with, an acidification of the medium. The strong acid deprotonates to peroxynitrite (ONOO^–^) at pK*_*a*_* values of 6.5–6.8 with the equilibrium reaction described by Equation (1).


$$\text{O}\,\text{N}\,\text{O}\,{\text{O}}^{-}+{\text{H}}^{+}↔\text{O}\,\text{N}\,\text{O}\,\text{O}\,\text{H}$$ (1)

The chemical and biological properties of ONOO^–^ and ONOOH have been presented in various publications due to their prominence as reactive intermediates in the atmosphere, in the human body, and in antimicrobial biochemistry (Goldstein et al., 2005; Koppenol et al., 2012; Loegager and Sehested, 1993; Pfeiffer et al., 1997). For example, reaction pathways and the importance of ONOO^–^ and ONOOH for the antimicrobial effect of PTM have been described in detail by Lukeš et al. (2015). ONOO^–^ and ONOOH are formed from the educts of H_2_O_2_ and $\text{N}\,{\text{O}}_{2}^{-}$, which are provided by a discharge in contact with water, according to Equations (2–4).


$${\text{H}}_{2}\,{\text{O}}_{2}+{\text{H}}_{3}\,{\text{O}}^{+}↔{\text{H}}_{3}\,{\text{O}}_{2}^{+}+{\text{H}}_{2}\,\text{O}$$ (2)

$$\text{N}\,{\text{O}}_{2}^{-}+{\text{H}}_{3}\,{\text{O}}^{+}↔\text{H}\,\text{N}\,{\text{O}}_{2}+{\text{H}}_{2}\,\text{O}$$ (3)

$${\text{H}}_{3}\,{\text{O}}_{2}^{+}+\text{H}\,\text{N}\,{\text{O}}_{2}↔\text{O}\,\text{N}\,\text{O}\,\text{O}\,\text{H}+{\text{H}}_{3}\,{\text{O}}^{+}$$ (4)

However, ONOOH is unstable in an acidic environment and has a lifetime of less than 1 s (0.8 s). Therefore, despite a strong antimicrobial potential, it is debatable if it is the primary agent responsible for the microbial inactivation provided by a plasma-treated liquid. However, continuous formation of this compound is plausible as long as abundant concentrations of its precursors are available or are continuously provided. The aim of the present study was to develop a detailed understanding of these possibilities, and in particular how the time course of peroxynitrous acid generation relates to the inactivation of bacteria. Reaction rates and the development of ONOOH concentrations depend in a crucial manner on pH values (Anbar and Taube, 1954; Damschen and Robbin Martin, 1983; Halfpenny and Robinson, 1952; Vione et al., 2003). The initial condition of the treated liquid, particularly its buffer capacity, must be taken into account. Consequently, the antimicrobial efficacy of plasma-treated tap water and distilled water strongly differ (Schmidt et al., 2019). In both cases, acidification through plasma treatment occurs due to the generation of HNO_2_/HNO_3_, and the conversion from $\text{N}\,{\text{O}}_{2}^{-}$ to $\text{N}\,{\text{O}}_{3}^{-}$ commences according to reactions described by Equations (5) and (6).


$$\text{O}\,\text{N}\,\text{O}\,\text{O}\,\text{H}↔\text{H}\,\text{N}\,{\text{O}}_{3}+\text{N}\,{\text{O}}_{3}^{-}+{\text{H}}^{+}$$ (5)

$$\text{O}\,\text{N}\,\text{O}\,\text{O}\,\text{H}↔\text{O}\,{\text{H}}^{-}+\text{N}\,{\text{O}}_{2}^{-}↔\text{N}\,{\text{O}}_{3}^{-}+{\text{H}}^{+}$$ (6)

In general, a lower pH value is advantageous for the formation of peroxynitrite and peroxynitrous acid. Tap water exhibits a certain buffer capacity, which limits acidification, whereas distilled water possesses little or no buffer capacity. However, the effective buffer capacity of tap water varies between locations depending on its mineral composition. A more systematic assessment can therefore be achieved by plasma exposure of defined buffer solutions. Independent of the water matrix, plasma treatment time is a key parameter governing the formation of H_2_O_2_, HNO_2_, and HNO_3_ and their subsequent conversion to ONOO^–^ and ONOOH. In aqueous systems, treatment and exposure times are directly linked to antimicrobial efficacy: longer times generally promote stronger acidification, higher concentrations of reactive species, and increased inactivation efficiency (Bakovsky et al., 2016; Kinandana et al., 2018). In contrast, several studies reported almost no inactivation in plasma-treated buffer solutions, which was attributed to their high buffer capacity preventing sufficient acidification and thus limiting the reactivity of plasma-generated species. Ikawa et al. showed that hardly any inactivation occurred above a pH value of 4.7, while effective inactivation required lower pH values (Ikawa et al., 2010). Similarly, Baik et al. observed pronounced inactivation when the pH of the treated solution was adjusted to 3.5 (Baik et al., 2013). Naitali et al. further demonstrated that neutralized or buffered plasma-treated water did not result in bacterial inactivation (Naitali et al., 2010).

Recent applied studies have further explored the antimicrobial efficacy of plasma-treated liquids under practical and food-related conditions and reported substantial variability in microbial inactivation depending on treatment configuration and physicochemical parameters (Choi et al., 2019; Fina et al., 2024; Xiang et al., 2019). In particular, temperature, solution composition, and buffering capacity have been identified as critical factors influencing reactive species chemistry and antimicrobial outcomes. Moreover, comparative investigations revealed pronounced differences in susceptibility between vegetative bacteria and bacterial spores, indicating that distinct inactivation mechanisms may be involved. Despite these advances, a quantitative mechanistic framework linking reactive species kinetics—especially peroxynitrous acid formation and decay—to microbial inactivation efficiency remains incomplete.

Building on these findings, the present study addresses the role of peroxynitrous acid in the antimicrobial activity of plasma-treated aqueous solutions. It is hypothesized that ONOOH represents the decisive inactivation agent and that its formation is governed by the buffer capacity of the plasma-treated medium. Consequently, it is necessary to distinguish potential inactivation effects arising from other plasma-generated reactive species or mechanisms from those specifically attributable to peroxynitrite and peroxynitrous acid. To this end, the temporal evolution of relevant reactive species was quantified and related to the inactivation of vegetative bacterial cells and bacterial spores. Within this framework, it was observed that direct plasma treatment alone was insufficient to achieve effective inactivation under the applied experimental conditions, indicating that HNO_2_, HNO_3_, H_2_O_2_, and ONOO^–^ alone do not constitute the primary decontamination agents.

Therefore, complementary experiments focused on plasma treatment of buffer solutions designed to promote the formation of high concentrations of peroxynitrous acid. The influence of buffer capacity on the accumulation of reactive species in plasma-treated media (PTM) was monitored by in-situ UV spectrometry. By systematically adjusting the buffer capacity, conditions leading to maximal inactivation were identified. To validate the reaction kinetics induced by plasma treatment, an analogous liquid chemical system was employed, in which H_2_O_2_ and NO_2_^–^ solutions of defined concentrations and pH values—derived from the plasma experiments—were mixed and assessed for antimicrobial activity. These experiments were supported by numerical simulations describing the time-dependent development of ONOOH concentrations, which are not directly accessible experimentally. In particular, the reaction pathways described in Equations (3–6) were investigated. The antimicrobial effects observed for plasma-treated liquids and their chemical analogues were comparable, yielding log_10_ reductions of up to 5.78 for *Escherichia coli* and 3.78 for *Bacillus atrophaeus* spores. In contrast, plasma-treated tap water resulted in log_10_ reductions of 3.84 for *E. coli* and 0.28 for *B. atrophaeus* spores. Notably, increasing plasma treatment or bacterial exposure times did not necessarily enhance inactivation, suggesting limitations in the achievable or sustainable concentrations of ONOOH.

## 2 Materials and methods

### 2.1 Experimental setup

**FIGURE 1:**
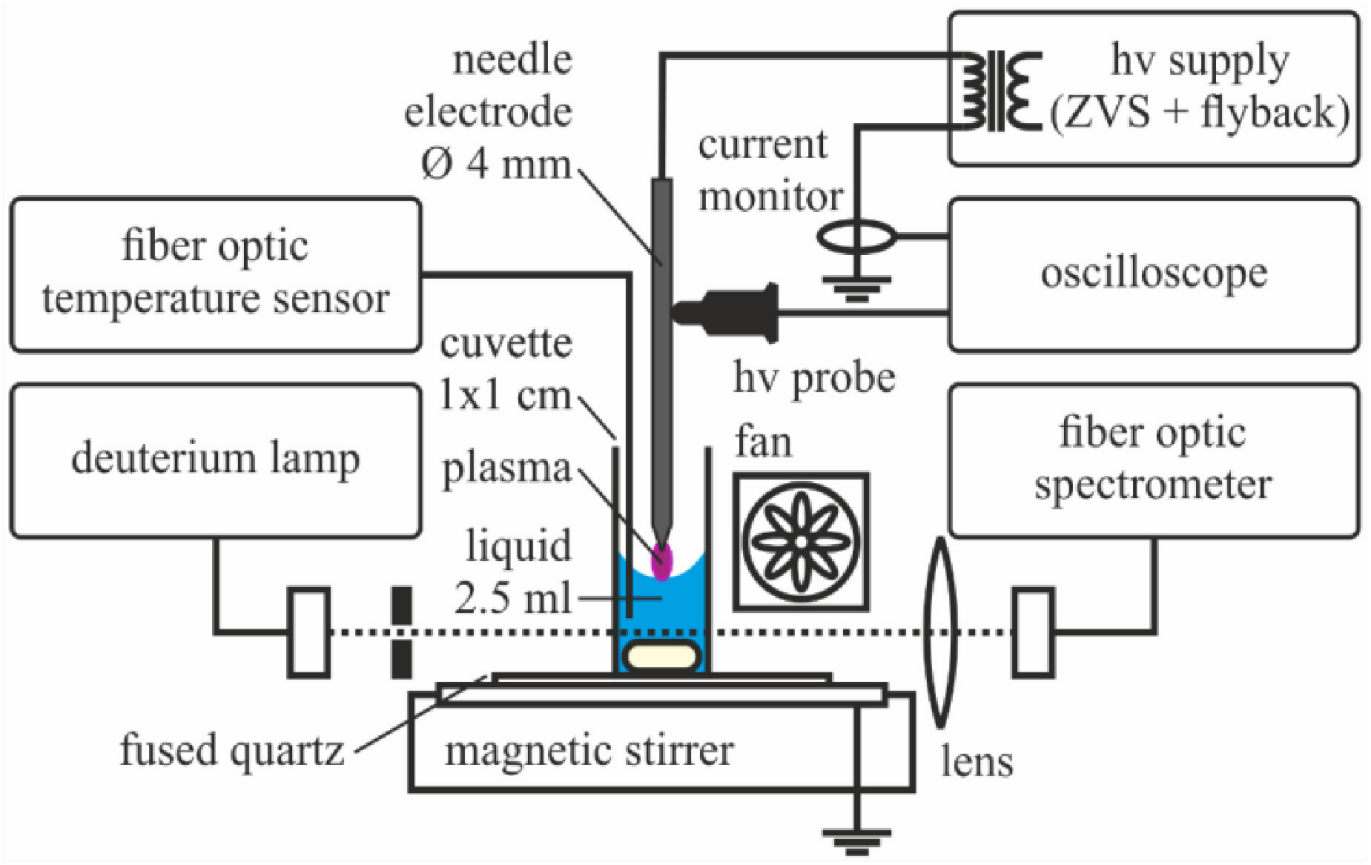
Principle of the experiments for the exposure of liquid to plasma together with components of the optical and electrical diagnostics.

Aqueous solutions with a volume of 2.5 mL were exposed to plasma from an electrical discharge between the solution and a tungsten electrode in ambient air as shown in Figure 1.

A solution of 2.5 mL was contained in a 1 × 1 cm^2^ optical cuvette and continuously mixed with a magnetic stirrer (neoLab D6010, neoLab Migge GmbH, Heidelberg, Germany). A plasma was established by an electrical discharge between the surface of the liquid and the tip of a tungsten needle electrode positioned at the center of the cuvette. The needle electrode had a diameter of 4 mm. The distance between the tungsten electrode and the liquid surface was adjusted to approximately 3 mm and maintained with a millimeter screw during plasma treatments to compensate for liquid evaporation caused by surface heating. The percentage loss of liquid volume following plasma treatment was approximately 20% after 1 h and 40% after 6 h. The remaining volume after treatment was measured and considered in the calculation of bacterial counts in the microbial inactivation experiments.

The plasma source operated as an atmospheric-pressure needle-to-liquid discharge in ambient air. The tungsten needle electrode served as the powered electrode, while the liquid surface acted as the counter electrode. Under the applied high-voltage excitation, a filamentary corona-type discharge was established at the gas–liquid interface.

Discharges were initiated with an oscillating high voltage at a frequency of 10 kHz and peak-to-peak amplitudes of approximately 20 kV, as determined from voltage probe measurements. An operating time of 3 s was followed by a pause of 3 s.

To determine the electrical power of the plasma source, simultaneous voltage and current measurements were performed. The high voltage was measured using a high-voltage probe (P6015A, Tektronix, Beaverton, OR, United States) connected to the tungsten electrode and referenced to ground. The discharge current was recorded using a current transformer (CT-F2.5-BNC, PEM, Nottingham, United Kingdom). Both signals were acquired with a digital oscilloscope (DPO 4104, Tektronix, Beaverton, OR, United States).

**FIGURE 2:**
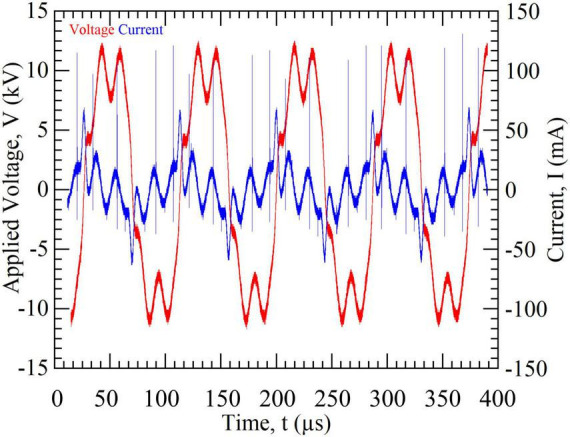
Voltage and current oscillograms of the plasma discharge recorded during operation above the liquid surface. The applied oscillating high voltage (red) with peak-to-peak amplitudes of approximately 20 kV generates pulsed discharge currents (blue) characterized by superimposed narrow current peaks. These peaks reflect filamentary microdischarge events typical of an atmospheric-pressure needle-to-liquid corona discharge at the gas–liquid interface.

During plasma treatment of the buffer solutions, voltage and current were recorded at regular intervals of 5–10 min within the 3-s active discharge phase. A representative oscillogram is shown in Figure 2. The accuracy of the current measurements is limited by the bandwidth of the current probe (500 MHz), resulting in a partial attenuation of high-frequency signal components. Nevertheless, the recorded voltage–current characteristics are sufficient to reliably describe the discharge behavior of the plasma source. The narrow filamentary peaks visible in the current signal do not originate from individual plasma filaments at the electrode–liquid interface but are attributed to partial discharges within the high-voltage circuitry and cable connections.

The instantaneous discharge power was calculated from the product of voltage and current waveforms. Averaging the power over multiple oscillation periods yielded the active electrical power of the discharge. Based on five independent measurements, an average power dissipation of approximately 1.1 W was determined under the applied operating conditions.

The temperature was monitored using a fiber-optic temperature sensor (TS5/1.0, Optocon, Dresden, Germany). The bulk liquid temperature did not exceed 32 °C even during extended treatment durations of up to 6 h.

Pertinent reactive species in the liquid were determined by UV absorption spectroscopy using focused illumination from a deuterium lamp (DH-2000, Ocean Optics, Dunedin, FL, United States). The spectra were recorded with a fiber-optic spectrometer (AvaSpec Dual, Avantes, Apeldoorn, The Netherlands). Ozone generated during plasma operation, which could interfere with absorbance measurements of the reactive species of interest, was removed using a fan (4715KL-04W-B40, NMB Technologies).

### 2.2 UV-spectrometric measurements

Light absorption measurements were conducted, in a spectral range of 200–450 nm, to determine respective concentrations of different chemical species. Since no monochromater was needed, the entire spectral range of the light source could be recorded simultaneously at a high temporal resolution down to 50 ms to follow any changes in concentrations with treatment time. The concentration of a detectable chemical compound was quantified for different time points from the observed light absorption according to Beer-Lambert’s law (Equation 7).


$$\text{A}\,\left(\nu \right)=-\text{ln}\frac{\text{I}\,\left(\nu \right)}{{\text{I}}_{0}\,\left(\nu \right)}=\sum {\text{n}}_{\text{i}}\cdot \sigma_{\text{i}}\cdot \text{d}$$ (7)

The absorbance, A, at a specific wavelength, ν, is related to the particle density, *n*_*i*_, of the particle species *i*, which is absorbing the light. The absorbance depends on the respective absorption cross-section, *σ_*i*_*, together with the absorption length, *d*, along the light path (specifically the width of the cuvette). Usually, photometric measurements are carried out at a fixed wavelength. Only data of effective cross-sections for a certain wavelength are available in literature, corresponding to the absorption peak of a particular molecule. Using this information, the individual contribution of specific species to the entire absorption spectrum was evaluated, which was recorded over an extended range of wavelengths to ensure relevant absorption lines were included. This approach was applied to determine the concentrations of H_2_O_2_, $\text{N}\,{\text{O}}_{2}^{-}$, $\text{N}\,{\text{O}}_{3}^{-}$, and HNO_2_ simultaneously.

### 2.3 Microorganisms and culture conditions

Inactivation studies were conducted with *Escherichia coli* K-12 DSM 11250/NCTC 10538 (vegetative cells; DSM-German Collection of Microorganisms and Cell Cultures, NCTC-National Collection of Type Cultures) or spores (dormant cells) of *Bacillus atrophaeus* DSM 675/ATCC 9372 (ATCC-American Type Culture Collection).

*E. coli* was cultured on soybean-casein digest (CASO) agar plates (Carl Roth GmbH & Co. KG, Karlsruhe, Germany). After an incubation of 24 h at 37°C, the agar plates were stored at 8°C.

For the experiments, *E. coli* were cultured in 20 mL of CASO broth (Carl Roth GmbH & Co. KG, Karlsruhe, Germany). After an incubation time of 24 h at 37°C, 10 mL of the culture was centrifuged (4,700 rpm; Heraeus Multifuge 1S, Thermo Fisher Scientific, Waltham, Massachusetts, United States) for 5 min. The supernatant was discarded and the microorganisms were suspended in 10 mL of saline solution (0.85% NaCl), which resulted in a total viable count of 1–3⋅10^9^ cfu/mL (colony forming units/mL, stock suspension). The vegetative cells of *B. atrophaeus* were obtained as vacuum dried culture. To gain spores of *B. atrophaeus*, the vegetative cells were cultivated on agar, which supported sporulation, for 7 days at 35°C. The sporulation agar consisted of 8.0 g of CASO broth, 4.0 g yeast extract, 20.0 g agar agar, and 0.03 g manganese sulfate (Merck KGaA, Darmstadt, Germany; Carl Roth GmbH & Co.KG, Karlsruhe, Germany). After incubation, the sporulation layer on the surface was removed using 10 mL of 0.85% NaCl solution and a Drigalski spatula. Subsequently, the material from two plates was transferred into tubes which were centrifuged twice at 5,000 rpm for 5 min. The resulting pellet was suspended in 0.85% NaCl and centrifuged again. Afterwards the pellet was dissolved in 20 mL deionized water. The spore suspension resulted in a total viable count of 1–3⋅10^9^ cfu/mL (stock suspension).

### 2.4 Plasma treatment process and inactivation studies

#### 2.4.1 Inactivation tests with plasma-treated media (indirect plasma treatment)

For the assessment of bacterial inactivation by plasma-treated media, plasma treatment time and exposure time needed to be distinguished from each other. Plasma treatment time describes the time that a solution is exposed to the plasma (treated) as shown in Figure 1. In contrast, the exposure time describes the time that bacteria remained in a plasma-treated solution (usually after the plasma treatment). Buffer solutions of different concentrations (dipotassium hydrogen phosphate (K_2_HPO_4_), Carl Roth GmbH & Co.KG, Karlsruhe, Germany) or tap water, at a volume of 2.5 mL, were treated with plasma for different durations from 30 to 281 min, depending on the experimental series. After plasma treatment, 10 μL of the bacterial stock solution (*B. atropheaus* spores or *E. coli*) were added to the treated solution (consequently 1–2⋅10^7^ cfu mL^−1^ during exposure experiments). For the determination of the viable cell count these samples were serially diluted in CASO broth and plated (five dilution levels: undiluted, 1:10, 1:100, 1:1,000, and 1:10,000). (Dilution 1:10: 100 μL of the bacteria-containing solution was mixed with 900 μL of CASO broth). Afterwards, 50 μL of the bacteria-containing CASO broth were plated immediately (within maximum 5 min) were plated per dilution using an automated spiral plater (Eddy Jet 2W, I&L Biosystem GmbH, Königswinter, Germany). This device applies the inoculum in a defined spiral pattern with continuously decreasing volume, enabling quantitative cfu determination across the plated dilution range (Gilchrist et al., 1973).

The agar plates were incubated at 30°C (for *B. atropheaus* spores) or 37°C (for *E. coli*) for 16 h.

The subsequent counting of the colonies was conducted with a colony counter (Flash & Go, I&L Biosystems, Königswinter, Germany). The control experiments were performed by employing untreated buffer solutions or tap water without plasma treatment according to the procedure described above.

The evaluation of the viable cell count reduction was depicted by the calculation of the reduction factor according to Equation 8.


logreduction10=log(N)control10-log(N)treatment10 (8)

where N*_*control*_* and N*_*treatment*_* indicate the number of colony-forming units (cfu) of the control and of the treated microorganisms, respectively.

All inactivation experiments were conducted as three independent biological replicates, with three technical replicates plated per dilution level. Results are reported as mean ± SD.

#### 2.4.2 Inactivation tests for direct plasma treatment

Different concentrations of K_2_HPO_4_ in solution or tap water, both containing bacteria, were treated directly with plasma to verify the efficacy of this approach. Therefore, 2.5 mL of the K_2_HPO_4_ solutions (5 mM and 30 mM) or tap water were pipetted into the 1 × 1 cm^2^ optical cuvette and inoculated with 10 μL of the bacterial stock solution (*B. atropheaus* spores or *E. coli*, subsection 2.3). The cuvette was placed in the experimental setup (Figure 1). The solution was treated with plasma for 3 min. The bacteria-containing solution was then further processed for the determination of the viable cell count as described in section 2.4.1.

#### 2.4.3 Control experiments to mimic the development of peroxynitrous acid and individual reactive species in solutions

To ascertain the antimicrobial efficacy of individual reactive species and to validate the observed inactivation in plasma-treated buffer solutions, a series of control experiments were conducted. These experiments included both solutions mimicking the concentrations of relevant species (for different plasma treatment times) and solutions with only individual species of known concentrations.

Initially, it was examined whether the buffer solutions themselves exhibited antimicrobial effects by testing 5 mM and 30 mM K_2_HPO_4_ solutions at room temperature (25°C) and under heated conditions (40°C). Additionally, analogous chemical experiments were performed with aqueous solutions of individual reactive species (Sol. #1–#4), as described in Table 1.

**TABLE 1:** Concentrations of the solutions used in the analogous chemical tests with aqueous solutions to study the antimicrobial effectiveness of individual species.

Solution no.	H_2_O_2_ (mM)	NO_2_^–^ (mM)	NO_3_^–^ (mM)	NO_2_ (mM), pH 3.2	Temperature
Sol. #1	45	0	0	0	25°C/40°C
Sol. #2	0	16	0	0	25°C/40°C
Sol. #3	0	0	53	0	25°C/40°C
Sol. #4	0	0	0	5	25°C/40°C

For these experiments, 990 μL of the respective aqueous solution was combined with 10 μL of bacterial stock solution (*B. atrophaeus* spores), initiating a 90 min exposure period. The viable cell count was determined, following the method described in subsection 2.4.1. Experiments were performed at room temperature (25°C) and at 40°C to assess the possible impact of temperature. Furthermore, a mixture of H_2_O_2_ (20 mM) and NO_2_^–^ (10 mM) solutions (Sol. #5) was prepared and heated.

To evaluate the antimicrobial activity, *B. atrophaeus* spores were exposed to these solutions. The experimental setup involved pipetting 495 μL of NaNO_2_ solution into an Eppendorf tube, followed by the addition of 10 μL of bacterial stock solution. Subsequently, 495 μL of the H_2_O_2_ solution was added to initiate a 90 min exposure period. After exposure, 100 μL of the solution was withdrawn and added to 900 μL of CASO broth to halt the chemical reaction between $\text{N}\,{\text{O}}_{2}^{-}$ and H_2_O_2_. The viable cell count was determined, following the method described in section 2.4.1.

The solutions used, denoted as Sol. #5-#11, were prepared according to Table 2 to mimic the concentration profiles for peroxynitrous acid formation observed during plasma treatment of K_2_HPO_4_ buffer solutions.

**TABLE 2:** Composition of solutions that were used to mimic the development of the production of ONOOH from NO_2_^–^ and H_2_O_2_ that was observed for the plasma treatment of K_2_HPO_4_ buffer solutions.

Solution no.	H_2_O_2_ (mM)	NO_2_^–^ (mM)	K_2_HPO_4_ (mM)	C_6_H_8_O_7_ (mM)	Temperature
Sol. #5	20	10	97	80	25°C/40°C
Sol. #6	30	15	97	80	25°C
Sol. #7	40	20	194	160	25°C
Sol. #8	60	30	291	225	25°C
Sol. #9	100	50	485	300	25°C
Sol. #10	200	100	970	365	25°C
Sol. #11	200	0	0	0	25°C

To reach a defined pH value of 4, a specific procedure was followed. For instance, sodium nitrite (NaNO_2_) and H_2_O_2_ solutions were mixed with K_2_HPO_4_ and citric acid (C_6_H_8_O_7_) to achieve the desired pH. The mixture was formulated to mimic the formation and degradation rates of ONOOH observed in the plasma-treated 30 mM K_2_HPO_4_ solution over a 90-min exposure time. Sodium nitrite, hydrogen peroxide (33%), and anhydrous citric acid (all from Carl Roth GmbH & Co.KG, Karlsruhe, Germany) were dissolved in deionized water. The antimicrobial efficacy was tested using the same experimental procedures.

## 3 Results

There is broad consensus that the antimicrobial efficiency of plasma-treated aqueous solutions is associated with the formation of reactive species. The aim of our work was to investigate the hypothesis that inactivation is dominated by peroxynitrous acid (ONOOH). The compound is formed in aqueous solutions from H_2_O_2_ and $\text{N}\,{\text{O}}_{2}^{-}$ (Equations 2–4) and can accumulate to elevated concentrations that are maintained for at least seconds to minutes at acidic conditions. Only a low solution pH enables the accumulation of ONOOH; otherwise, it is present only at low concentrations. In the first step, we determined and characterized the conditions and time courses that produce sustained high concentrations of ONOOH from the reaction of H_2_O_2_ and $\text{N}\,{\text{O}}_{2}^{-}$ in K_2_HPO_4_ solutions with different buffer capacities and, to some extent, in tap water (which has some inherent buffer capacity). Unfortunately, the transient nature of ONOOH prevented direct observation or quantification. Respective concentrations were generally too low for measurements by UV spectrometry. However, different phases of ONOOH generation and degradation were accessible from observations of the concentrations of H_2_O_2_ and $\text{N}\,{\text{O}}_{2}^{-}$, which corresponded with the inactivation of vegetative bacterial cells and dormant spores. Reaction kinetics were dependent on pH and a sufficiently acidic environment was necessary for the initial substances to react with each other and for ONOOH to be formed. However, when microorganisms were exposed too early during the formation process, concentrations sufficient for effective inactivation had not yet been reached. Likewise, the antimicrobial properties of the solution had expired when ONOOH was already decomposed. Subsequent experiments were conducted to confirm the proposed inactivation by ONOOH. In particular, we showed that direct exposure of the media to plasma, which did not respect the conditions and time course needed for the accumulation of ONOOH, resulted in only moderate inactivation. Accordingly, short-lived species that were provided directly by the plasma (for example atomic oxygen), as well as other direct effects such as UV exposure or elevated temperature, played only a minor role. Likewise, precursors of ONOOH (high concentrations of H_2_O_2_ and $\text{N}\,{\text{O}}_{2}^{-}$ alone) did not cause significant inactivation, even at elevated temperatures under the chosen test conditions.

### 3.1 Peroxynitrous acid formation and decay determine inactivation windows

Our study pursued the general hypothesis that sustainable concentrations of peroxynitrous acid are necessary to achieve high inactivation rates for bacteria and spores. High concentrations require an accumulation of the reactants for a sufficiently long time, which, in turn, necessitates the control of pH. To determine this, two solutions with different concentrations of K_2_HPO_4_ (5 mM and 30 mM), which had different buffer capacities, were treated with plasma, and the concentrations of the respective reactive species were measured over the treatment period.

#### 3.1.1 Sustained peroxynitrous acid concentrations depend on buffer concentrations

**FIGURE 3:**
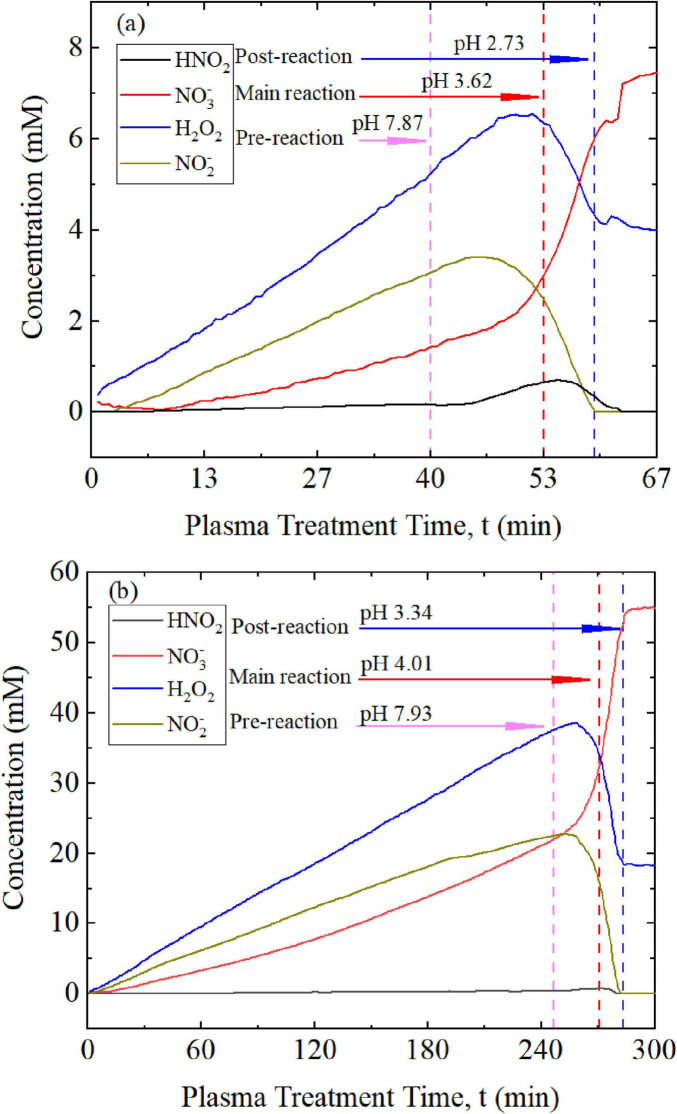
Concentrations of H_2_O_2_, $\text{N}\,{\text{O}}_{2}^{-}$, $\text{N}\,{\text{O}}_{3}^{-}$, and HNO_2_ in a K_2_HPO_4_ buffer solutions of **(a)** 5 mM and **(b)** 30 mM during plasma treatment. Concentrations of H_2_O_2_ and $\text{N}\,{\text{O}}_{2}^{-}$ initially increase almost linearly, then decline, indicating an increasing rate of conversion to ONOOH. Concurrently, the formation of the intermediate HNO_2_ and the decomposition of ONOOH into $\text{N}\,{\text{O}}_{3}^{-}$ is accelerated.

Figure 3 shows the development in concentrations of the relevant species (H_2_O_2_, $\text{N}\,{\text{O}}_{2}^{-}$, $\text{N}\,{\text{O}}_{3}^{-}$, and HNO_2_) as a function of plasma treatment time in 5 mM and 30 mM K_2_HPO_4_ buffer solutions.

However, the onset of this decay is apparent even as H_2_O_2_ and $\text{N}\,{\text{O}}_{2}^{-}$ concentrations rise. Accordingly, three windows are defined: a pre-reaction window (phase 1; end marked by the pink dashed line), when the decomposition of ONOOH is negligible; a main reaction window (phase 2; end marked by the red dashed line), when ONOOH formation dominates; and a post-reaction window (phase 3; end marked by the blue dashed line), when decomposition predominates. The three phases are also associated with different pH values. An initial buffer concentration six times higher produced proportionally higher peak concentrations of H_2_O_2_ and $\text{N}\,{\text{O}}_{2}^{-}$, which both took about six times longer to achieve.

During the plasma treatment, three main phases in the development of the concentrations of the relevant species were identified. The first phase (pre-reaction) involved the introduction and accumulation of reactive species such as H_2_O_2_ and $\text{N}\,{\text{O}}_{2}^{-}$, and already some$\text{N}\,{\text{O}}_{3}^{-}$; the latter species may have been produced directly by the plasma or formed as a decomposition product of ONOOH (Equations 3–6). The end of the phase is characterized by a distinct decrease in concentration of H_2_O_2_ and $\text{N}\,{\text{O}}_{2}^{-}$. The subsequent second phase (main reaction) was distinguished by an increasing conversion of $\text{N}\,{\text{O}}_{2}^{-}$ and H_2_O_2_ into ONOOH. As the solution acidified and the buffer capacity was exhausted, ONOOH formation (Equations 2–4) dominated, explaining the observed decreases in the reactant species. Correspondingly, elevated HNO_2_ concentrations were also detected. Moreover, the concentration of $\text{N}\,{\text{O}}_{3}^{-}$ increased as well, which is reflected in the steeper slope, indicating that ONOOH was progressively decomposed. The second phase ended when the $\text{N}\,{\text{O}}_{2}^{-}$ concentration approached zero and could no longer support the formation of ONOOH. In the ensuing third phase (post-reaction), the only remaining species were H_2_O_2_ and $\text{N}\,{\text{O}}_{3}^{-}$; only $\text{N}\,{\text{O}}_{3}^{-}$ continued to be carried into the liquid, while H_2_O_2_ remained at a constant level.

The development of the reactive species concentrations was qualitatively the same for both the 5 mM and 30 mM K_2_HPO_4_ buffer concentrations. However, clear differences in the absolute values and the time course of concentration developments were determined. The highest concentration of H_2_O_2_ was 6.2 mM in the 5 mM buffer solution compared to 37 mM in the 30 mM buffer solution—about 6 times higher. The $\text{N}\,{\text{O}}_{2}^{-}$ concentrations were with 20 mM about eight times higher for the high concentration buffer solution compared to 2.5 mM found for the lower buffer concentration. The same difference was observed for $\text{N}\,{\text{O}}_{3}^{-}$ with values of 7.2 mM in the 5 mM buffer and 55 mM in the 30 mM buffer. The increase in the amount of $\text{N}\,{\text{O}}_{3}^{-}$ correlated with the concentration maxima of H_2_O_2_ and $\text{N}\,{\text{O}}_{2}^{-}$, which supports the assumption of a reaction pathway via the formation and degradation of ONOOH (Equations 2–6). Corresponding to the differences in initial buffer concentrations, different time courses were observed. A steep increase in the concentration occurred 35 min for the 5 mM buffer solution but only at 276 min in the 30 mM buffer solution, roughly 7–8 times longer. Differences in species concentrations and their temporal development roughly reflect the sixfold increase in K_2_HPO_4_ concentration between the two plasma-treated solutions. Of particular interest was the development of HNO_2_. In absolute terms, the maximum concentration of HNO_2_ was approximately 1 mM in both the 5 mM and 30 mMK_2_HPO_4_ solutions. However, this may only suggest the intermediate role of this compound. Consistent with this interpretation, the relative concentration of HNO_2_ decreased compared with other induced reactive species as the K_2_HPO_4_ concentration increased.

The main purpose of the different buffer concentrations was the response of the solution to acidification induced by the plasma treatment. Acidification was less pronounced in higher concentration buffer solutions and increased as buffer solution concentration decreased. This trend is shown by pH measurements taken at different times during the plasma treatment. The time points indicated in Figure 3 define the transition between the observed phases: before ONOOH formation, during ONOOH formation, and after ONOOH decomposition. Table 3 shows the pH values that were characteristic for the different phases. When the buffer capacity was exhausted, acidification was intensified by the conversion of $\text{N}\,{\text{O}}_{2}^{-}$ to HNO_2_ and the formation of HNO_3_. The pH value eventually obtained in the post-reaction phase did not change any further.

**TABLE 3:** Measured pH values at the end of each phase (see Figures 3, 4).

Medium	pH-value phase 1	pH-value phase 2	pH-value phase 3
5 mM K_2_HPO_2_ buffer	7.87	3.62	2.73
30 mM K_2_HPO_2_ buffer	7.93	4.01	3.34
Tap water	7.90	3.42	2.67

#### 3.1.2 Peroxynitrous acid reaction environment of tap water

**FIGURE 4:**
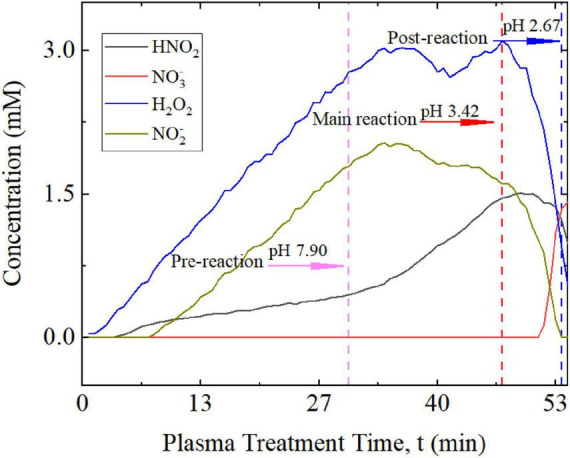
Concentrations of H_2_O_2_, $\text{N}\,{\text{O}}_{2}^{-}$, $\text{N}\,{\text{O}}_{3}^{-}$, and HNO_2_ in tap water during plasma treatment. Concentrations of H_2_O_2_ and $\text{N}\,{\text{O}}_{2}^{-}$ initially linearly increased before reaching a broad maximum, which was sustained for about 15 min, before dropping off. The decrease indicated an increasing conversion rate into ONOOH. Concurrently, formation of the intermediate HNO_2_ and the decomposition of ONOOH into $\text{N}\,{\text{O}}_{3}^{-}$ was accelerated. However, HNO_2_ was formed approximately at the start of the plasma treatment. Three windows are defined depending on the concentration development: a pre-reaction window (phase 1; end marked by the pink dashed line) when the decomposition of ONOOH is negligible; a main reaction window (phase 2; end marked by the red dashed line), when ONNOH formation dominates; and a post-reaction window (phase 3; end marked by the blue dashed line), when decomposition predominates. The three phases are also observed in plasma-treated buffers and are associated with different pH values.

Plasma-treated aqueous solutions used for the purpose of inactivating water borne pathogens do not usually include a dedicated buffer system. Instead, deionized water is often the basis for medical applications although many direct applications rely on mechanisms that are mediated by bodily fluids. The application of plasma-treated aqueous solutions as a disinfectant, such as for surface cleaning or in food hygiene, is mostly conducted with tap water. Tap water exhibits at least some buffer properties, which depend on specific characteristics that differ by source and location. The variety and complexity of waste water and process water is even larger than tap water, while deionized or distilled water are known to not have significant buffer characteristics. For comparison with the defined K_2_HPO_4_ buffer solutions that were studied, and with respect to the relevance for disinfection purposes, conditions for the formation of peroxynitrous acid were also studied with the local tap water available in Greifswald, Germany, as an instructive example. The tap water was treated with plasma in the same way as the K_2_HPO_4_ buffer solutions. Figure 4 shows the development of the relevant species, including H_2_O_2_, $\text{N}\,{\text{O}}_{2}^{-}$, HNO_2_ and $\text{N}\,{\text{O}}_{3}^{-}$.

In principle, the same qualitative development was observed in tap water as found in the defined buffer solutions, especially for H_2_O_2_ and $\text{N}\,{\text{O}}_{2}^{-}$. An initial linear increase in H_2_O_2_ and $\text{N}\,{\text{O}}_{2}^{-}$ concentrations peaked at about 3 and 1.7 mM, respectively. However, the maxima were less defined: the peaks were broader in time than those in the K_2_HPO_4_ buffer solutions, though they occurred on a similar time scale to the 5 mM buffer solution. The absolute values were similar but approximately twofold lower. The subsequent decrease in concentrations was considerably faster than the initial increase and was accompanied by rising $\text{N}\,{\text{O}}_{3}^{-}$ concentrations. $\text{N}\,{\text{O}}_{2}^{-}$ was no longer detectable after 53 min.

A notable difference was observed in the development of nitrous acid. HNO_2_ concentrations began rising at the start of plasma exposure but increased in speed once the highest concentrations had been reached for H_2_O_2_ and $\text{N}\,{\text{O}}_{2}^{-}$. Regardless, the maximum concentration of 1.5 mM was similar to the experiments with 5 mM and 30 mM buffer solutions. Altogether this suggests that tap water follows the same reaction mechanisms, including the intermediate nature of HNO_2_. Moreover, pre-reaction, main reaction, and post-reaction phases could be identified correspondingly with distinct pH values. However, with the weaker buffer capacity (in comparison to the 30 mM K_2_HPO_2_ buffer), acidification associated with plasma treatment and instigated chemical reactions progressed much faster, and the pH dropped to a slightly lower final value of 2.67 (Figures 3, 4 and Table 3).

#### 3.1.3 Confirmation of peroxynitrite reaction kinetics by modeling

**FIGURE 5:**
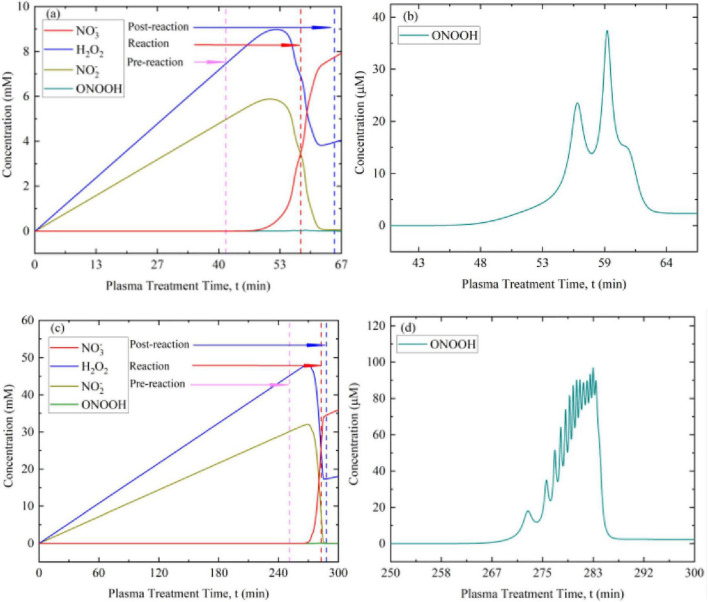
Concentration profiles of H_2_O_2_, NO_2_^−^, NO_3_^−^, and ONOOH in K_2_HPO_4_ buffered solutions of two buffer strengths. **(a)** Simulated concentration profiles for a K_2_HPO_4_ of 5 mM; **(b)** simulated ONOOH formation and decay within the active main reaction phase. **(c)**, **(d)** Corresponding results for 30 mM K_2_HPO_4_. The simulated plasma treatment introduces reactive species, including H_2_O_2_ and $\text{N}\,{\text{O}}_{2}^{-}$, as indicated by the almost linear increase. After reaching peak values, the rapid decline in concentrations of H_2_O_2_ and $\text{N}\,{\text{O}}_{2}^{-}$ signifies an increased conversion to ONOOH, although ONOOH concentrations remained about three orders of magnitude lower than of H_2_O_2_ and $\text{N}\,{\text{O}}_{2}^{-}$. However, the concentrations of these reactants influenced the equilibrium, tilting it toward ONOOH formation. The intermittent peaks in ONOOH concentrations signify periods of enhanced production, while the troughs represent intervals where degradation processes predominate. The cyclic nature of these fluctuations underscores the dynamic equilibrium existing between the various reactive species involved in ONOOH formation and degradation. The decomposition of ONOOH is associated with an increase in $\text{N}\,{\text{O}}_{3}^{-}$ concentrations. Temporal development and absolute concentrations are similar to the experimental observations (Figures 3a,b) and confirm the assumed underlying reaction kinetics.

The indirect assessment of ONOOH formation and decay kinetics through H_2_O_2_ and NO_2_^–^ measurements was validated by numerical simulations based on Equations 1–6. Species concentrations, c_*i*_, were determined by solving a system of ordinary differential equations:


dc/idt=G-L (9)

where *G* and *L* represent species-specific production and loss terms, respectively. Constant production rates for HNO_2_ and NO_2_^−^ were adopted from Vione et al. (2003), whereas rates for H_2_O_2_ and NO_2_^−^ were derived from UV data. Simulations were performed at a reference temperature of 25 °C and yielded excellent agreement with the experimental results (Figures 3–5), despite actually having a slightly higher measured solution temperature of about 32°C. The model confirmed that the consumption of HNO_2_, NO_2_^−^, and H_2_O_2_ is tightly coupled to ONOOH generation via the two key reactions:


$${\text{H}}_{2}\,{\text{O}}_{2}+{\text{H}}_{3}\,{\text{O}}^{+}↔{\text{H}}_{3}\,{\text{O}}_{2}^{+}+{\text{H}}_{2}\,\text{O}$$ (10)

$${\text{H}}_{3}\,{\text{O}}_{2}^{+}+\text{H}\,\text{N}\,{\text{O}}_{2}↔\text{O}\,\text{N}\,\text{O}\,\text{O}\,\text{H}+{\text{H}}_{3}\,{\text{O}}^{+}$$ (11)

The simulations revealed the same three distinct kinetic regimes observed experimentally: a pre-reaction window, where ONOOH has not yet formed; a main reaction window, dominated by ONOOH production; and a post-reaction window, characterized by intensified decomposition. The low simulated ONOOH concentrations confirm its absence in the UV spectra and highlight the added value of modeling.

The ONOOH concentration curve exhibits pronounced peaks and troughs, reflecting the dynamic equilibrium between concurrent formation and degradation pathways (Equations 9–11). Peaks mark periods of enhanced production, whereas troughs indicate dominant decay reactions. This cyclic behavior mirrors the complex, multistep interplay among H_2_O_2_, NO_2_^−^, HNO_2_, and ONOOH.

The simulations further showed that increasing the buffer concentration sixfold resulted in proportionally higher peak concentrations of H_2_O_2_ and NO_2_^−^ and extended the time to reach these maxima by approximately the same factor. Acidification, driven by the formation of HNO_2_ and HNO_3_ and the conversion of NO_2_^−^ to NO_3_^−^, was strongly modulated by buffer capacity. Higher buffering delayed acidification but prolonged the ONOOH production window. As acidification advanced, the equilibrium shifted toward ONOOH formation until reactant depletion initiated the degradation-dominated phase 3.

#### 3.1.4 Bactericidal and sporicidal efficacy depend on inactivation windows

**FIGURE 6:**
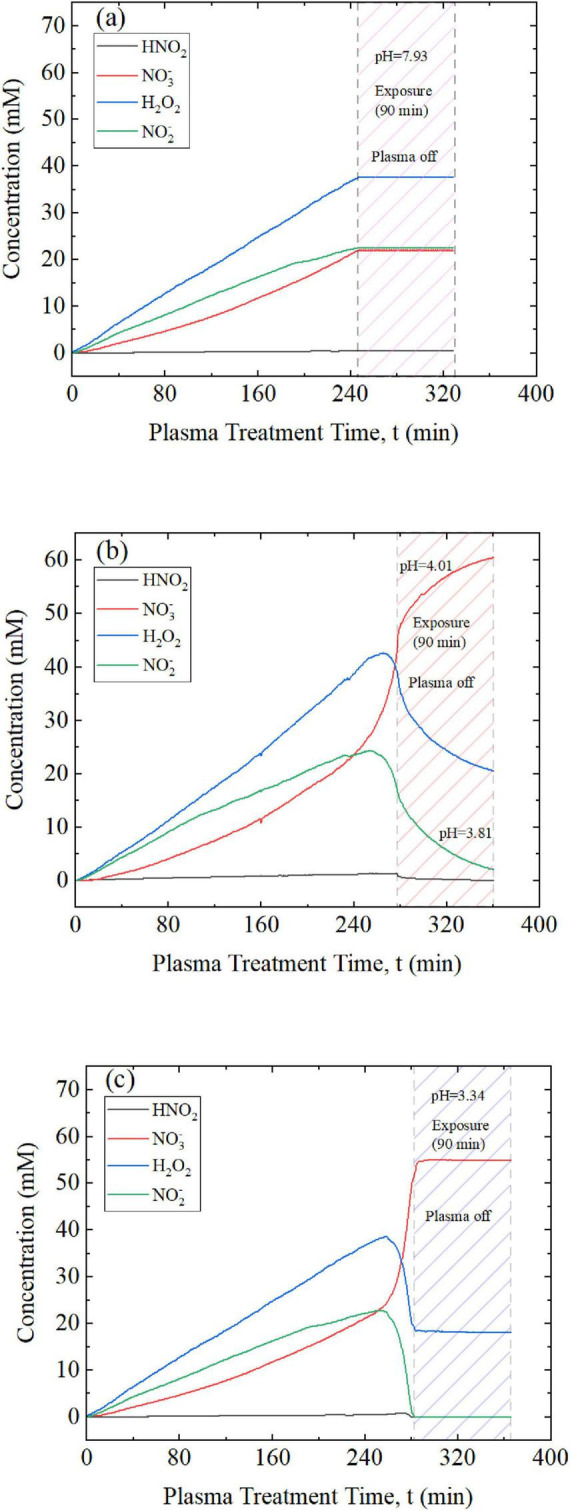
Concentrations of H_2_O_2_, $\text{N}\,{\text{O}}_{2}^{-}$, $\text{N}\,{\text{O}}_{3}^{-}$, and HNO_2_ in a 30 mM K_2_HPO_4_ buffer solution until plasma treatment was stopped and *B. atrophaeus* (dormant spores; concentration: 1–2⋅10^7^ cfu/mL) were added **(a)** during the pre-reaction window (phase 1; the pink shaded area highlights the phase in the exposure time period), **(b)** at the start of the main reaction window (phase 2; the red shaded area highlights the phase in the exposure time period), or **(c)** at the beginning of the post-reaction window (phase 3; the blue shaded area highlights the phase in the exposure time period). Respective end-points for the plasma treatment and times for the introduction of vegetative cells or dormant spores are indicated by the first vertical dashed line. In all three cases **(a–c)** the bacteria were exposed for 90 min to the plasma-treated suspension as indicated by the hashed areas and the second dashed line (as end point). In addition, the pH values for the three distinct exposure periods are presented.

Based on the observed development of the chemical species during plasma exposure, high production rates and concentrations of peroxynitrous acid were expected, particularly during the identified second phase of the reaction dynamic (Figures 3, 4). Following the pursued hypothesis, inactivation of microorganisms should be most effective during this main reaction phase. Conversely, in phase 1 significant amounts of ONOOH have not yet build up, while in phase 3 they have already been depleted. To validate the assumption, plasma treatment was stopped during the pre-reaction phase (phase 1), at the onset of the main reaction phase (phase 2), or at the start of the post-reaction phase (phase 3) and vegetative or dormant bacteria were suspended in the respective solutions (Figure 6). Therefore, stock solutions of *E. coli* or spores of *B. atrophaeus* (section 2.4.1) were mixed with a plasma-treated volume of 1.5 mL (5 or 30 mM K_2_HPO_4_ or tap water).

**FIGURE 7:**
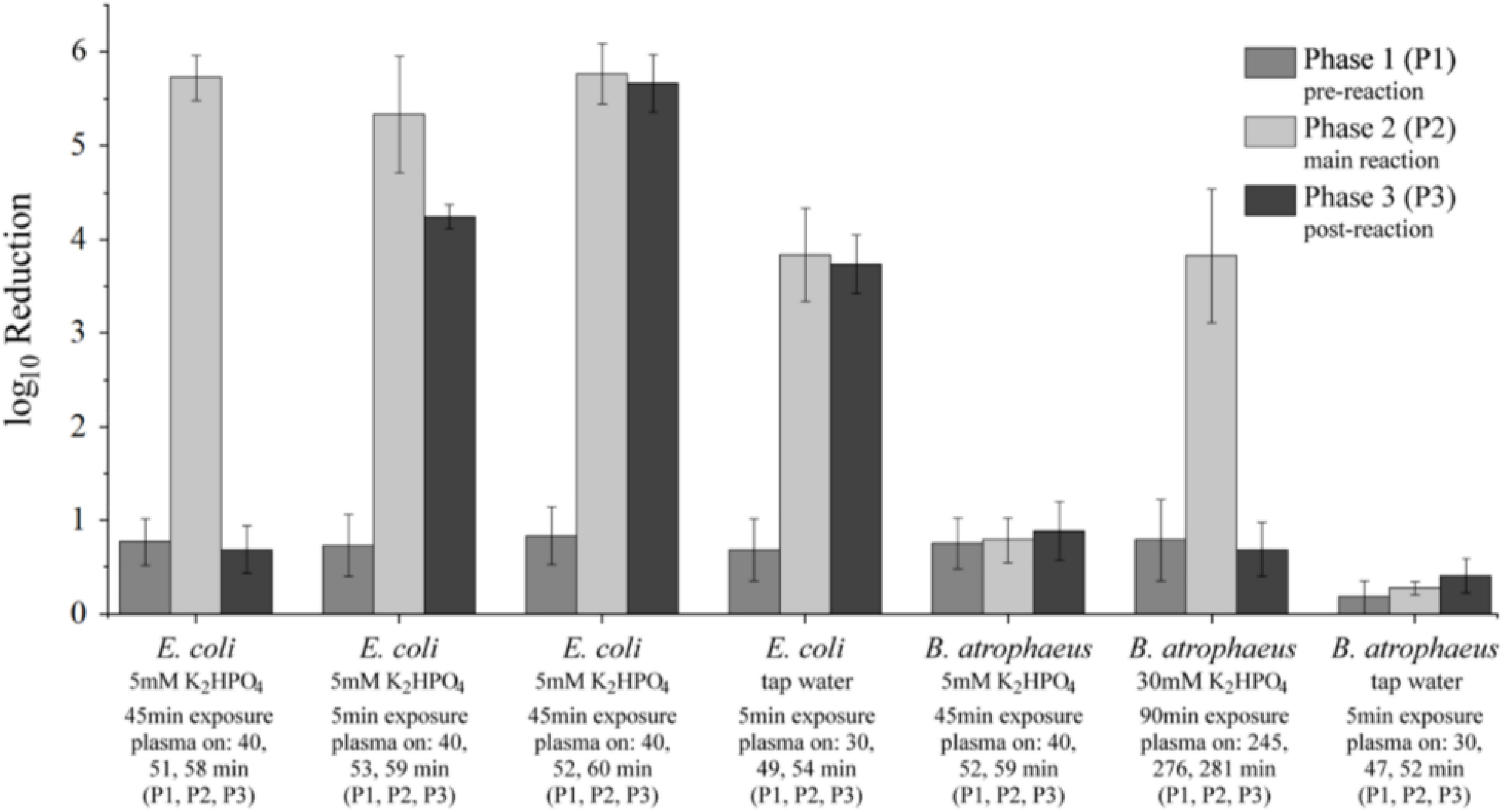
log_10_ reductions of *E. coli* (vegetative cells, 1–2 ⋅ 10^7^ cfu/mL) and *B. atrophaeus* (spores, 1–2 ⋅ 10^7^ cfu/mL) obtained during the three plasma-treatment phases: pre-reaction window (phase 1: P1, gray), main reaction window (phase 2: P2, light gray), and post-reaction window (phase 3: P3, dark gray). Data correspond to exposure conditions and log_10_ reductions that are summarized in Table 4 and illustrate the phase-dependent differences in bacterial inactivation efficiency. Significant inactivation of *E. coli* was observed in phase 2, both in the 5 mM buffer solution and in tap water, with reductions ranging from between four to six orders of magnitude. This level of inactivation also persisted in phase 3, indicating that factors present in this stage contributed to bacterial reduction (with the exception for a 3 min exposure time).

After the bacteria were added to the plasma-treated solutions, they were incubated for the defined exposure time. Afterwards, the viable cell count determined inactivation. The exposure time was defined with 90 min (Figure 6a) due to the previously determined complete decomposition of $\text{N}\,{\text{O}}_{2}^{-}$ when the plasma treatment had been stopped and after the buffer capacity for the K_2_HPO_4_ solutions of 5 mM or 30 mM was depleted. The associated pH value did then not change anymore and H_2_O_2_ and $\text{N}\,{\text{O}}_{3}^{-}$ persisted in stable concentrations in the solution (Figure 6c). This was also the case for the suspension of bacteria during the pre-reaction phase (Figure 6a) where the formation of ONOOH was impeded, since the buffer capacity was not yet exhausted. Conversely, the turnover of H_2_O_2_ and $\text{N}\,{\text{O}}_{2}^{-}$ during the main reaction phase (Figure 6b) still changed in concentration and led to a further acidification. Since the acidification of the 5 mM K_2_HPO_4_ solution progressed faster than the 30 mM K_2_HPO_4_ solution, the plasma treatment time could be shortened to approximately 50 min when the buffer capacity was exhausted and a direct degradation of $\text{N}\,{\text{O}}_{2}^{-}$ commenced. The time of exposure of bacteria could also be reduced to 45 min with respect to the results presented in Figure 6a. The buffer capacity of tap water was exhausted even faster and exposure times were limited to 5 min. The results for the inactivation of *E. coli* or spores of *B. atrophaeus*, depending on their exposure in different windows of the reaction chemistry for peroxynitrous acid, is summarized in Table 4 and illustrated in Figure 7.

**TABLE 4:** Inactivation (log_10_ reduction) of *E. coli* (vegetative cells; 3⋅10^7^ cfu/mL) and *B. atrophaeus* (dormant spores; concentration: 1–2⋅10^7^ cfu/mL) that were added at different phases of reactive species production during plasma treatment and exposed to the suspensions for different times.

Medium	Test organism	Exposure time in suspension (min)	Plasma treatment time phase 1/phase 2/phase 3 (min)	log_10_ reduction phase 1	log_10_ reduction phase 2	log_10_ reduction phase 3
5 mM K_2_HPO_4_	*E. coli*	3	40/51/58	0.77 ± 0.25	5.73 ± 0.24	0.69 ± 0.25
5 mM K_2_HPO_4_	*E. coli*	5	40/53/59	0.73 ± 0.33	5.34 ± 0.62	4,25 ± 0.13
5 mM K_2_HPO_4_	*E. coli*	45	40/52/60	0.84 ± 0.31	5.78 ± 0.32	5.67 ± 0.31
Tap Water	*E. coli*	5	30/49/54	0.69 ± 0.33	3.84 ± 0.5	3.73 ± 0.3
5 mM K_2_HPO_4_	*B. atrophaeus*	45	40/52/59	0.76 ± 0.27	0.79 ± 0.24	0.89 ± 0.31
30 mM K_2_HPO_4_	*B. atrophaeus*	90	245/276/281	0.79 ± 0.43	3.83 ± 0.71	0.69 ± 0.29
Tap water	*B. atrophaeus*	5	30/47/52	0.18 ± 0.18	0.28 ± 0.07	0.41 ± 0.18

Results are presented as the mean ± SD (standard deviation) for three repeated experiments.

In contrast, *B. atrophaeus* spores showed lower inactivation across the different phases. In phase 1, where ONOOH had not yet formed, and in phase 3, where ONOOH had already degraded, no significant microbial inactivation was observed (< 1 log_10_). However, for a K_2_HPO_4_ solution of 30 mM and an exposure time of 90 min in phase 2, an inactivation of four orders of magnitude was achieved, whereas inactivation in phase 3 remained marginal.

Plasma treatment leads to acidification of the buffer solution and a decrease in buffer capacity. Once this is used up, the formation and degradation mechanisms of ONOOH (Equations 2–6) are initiated. HNO_2_ is then formed, acidification increases, and the pH decreases. The third phase starts when $\text{N}\,{\text{O}}_{2}^{-}$ and HNO_2_ are completely degraded, as shown by their concentration curves dropping to zero, and thus ONOOH is completely decomposed. The result strongly supports the hypothesis that microbial inactivation by ONOOH is the most important mechanism when the buffer capacity is depleted and the reaction to peroxynitrous acid becomes dominant. The findings further emphasize the importance of the formation of ONOOH over acidification, as even in phase 3 there is no significant inactivation for *B. atrophaeus*, but the pH had already dropped significantly. Depending on the initial concentration of the buffer solutions, larger amounts of ONOOH could be accumulated.

### 3.2 Identification of relevant inactivation processes

The reaction conditions required to form and maintain sufficient amounts of peroxynitrous acid for an extended time can be controlled by the buffer capacity and the acidity of an aqueous solution. Accordingly, different formulations have been prepared and compared with tap water as the medium, which is for practical applications the most relevant. Microbial inactivation caused by these solutions themselves and during direct plasma treatment were investigated to distinguish if there are any effective compounds and mechanisms that are different from inactivation caused by ONOOH.

#### 3.2.1 Direct plasma treatments result in only minor inactivation

To explore possible inactivation of vegetative and dormant bacterial cells by mechanisms and processes associated with direct plasma exposure, the different aqueous solutions were inoculated with *E. coli* or *B. atrophaeus* spores and treated directly. Figure 7 shows the results of the experiments. The exposures resulted in a maximum reduction of 0.7 log_10_. Accordingly, species that were present only while the plasma impinged on the liquid, such as hydrated electrons, atomic oxygen, hydroxyl radicals (OH⋅), and more stable transient species (such as ${\text{O}}_{2}^{-}$), did not contribute significantly to the antimicrobial response. Additionally, the UV emissions may not be effective in these experiments. Although the direct interaction mechanisms may explain the limited inactivation observed, it is also possible that the beginning of peroxynitrous acid formation already played a role.

#### 3.2.2 Elevated hydrogen peroxide and nitrite concentrations result in only moderate inactivation

**FIGURE 8:**
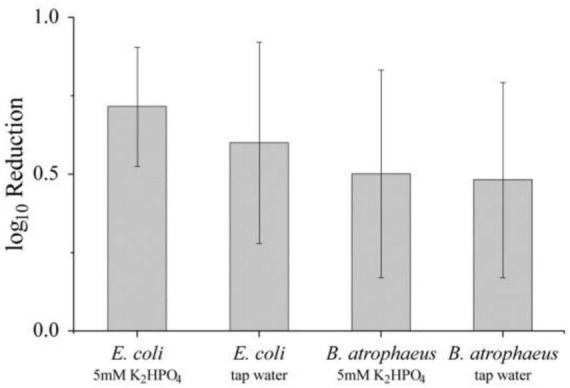
Inactivation of dormant *B. atrophaeus* spores (1–2⋅10^7^ cfu/mL) suspended in aqueous solutions at room temperature with concentrations of reactive species similar to those achieved during plasma treatment of a 30 mM K_2_HPO_4_ solution. Data correspond to exposure conditions and log_10_ reductions that are summarized for the different solutions in Tables 1, 2.

The most prominent long-lived species observed in our and other studies following reactions of short-lived species from the interaction of non-thermal plasma with water were H_2_O_2_ and $\text{N}\,{\text{O}}_{2}^{-}$. Both are antimicrobial agents and their individual potential for an inactivation was investigated. The results for dormant *B. atrophaeus* spores in the different solutions treated for 90 min are shown in Figure 8. *B. atrophaeus* spores were chosen for the control experiments because of their higher resilience compared to *E. coli*, ensuring robust testing conditions. The viable count before and after exposure was compared to the respective solution. Although Sol. #2 and #3 showed a reduction of about one order of magnitude—greater than that observed for direct exposures—the overall antimicrobial response remained rather moderate. Therefore, the individual contribution of H_2_O_2_ or $\text{N}\,{\text{O}}_{2}^{-}$ to inactivation were considered limited.

#### 3.2.3 Moderate temperature increase does not promote better inactivation

**FIGURE 9:**
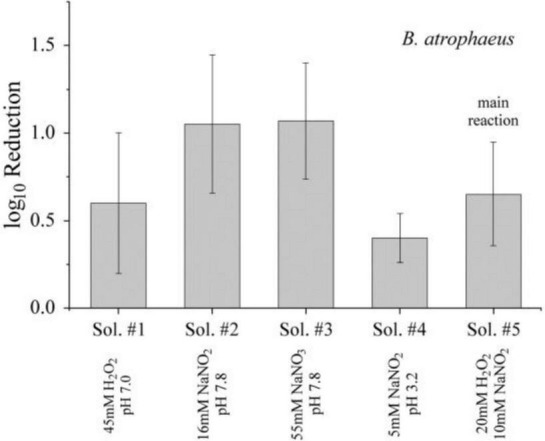
Inactivation of dormant *B. atrophaeus* spores (1–2⋅10^7^ cfu/mL) suspended in aqueous solutions containing individual or combined with concentrations of reactive species similar to those achieved during plasma treatment of a 30 mM K_2_HPO_4_ solution. All samples were incubated at 40 °C to evaluate the influence of elevated temperature on antimicrobial efficacy. Data correspond to exposure conditions and log_10_ reductions that are summarized for the different solutions in Tables 1, 2.

Plasma treatment is associated with an increase in temperature of the solutions. Therefore, it was conceivable that the higher temperatures could affect chemical reaction rates and inactivation kinetics. For a more detailed assessment of the latter, solutions were heated to 40°C before bacteria or spores were added (1–2⋅10^7^ cfu/mL). To investigate whether higher temperatures promote conversion to ONOOH, and thereby increase inactivation, we compared buffer solutions containing 5 mM and 30 mM K_2_HPO_4_. Additionally, a mixture of 20 mM H_2_O_2_ and 10 mM NO_2_^–^ (Sol. #5) was prepared and heated to assess its antimicrobial efficacy under elevated temperature conditions. The results of the inactivation experiments are presented in Figure 9. The outcomes are indistinguishable from the analogous chemical tests at room temperature (25°C) (Figure 8). Higher concentrations of the buffer or reactants did not result in a noticeable advantage. Consequently, a moderate temperature increase did not lead to higher inactivation.

#### 3.2.4 Concentrations of H_2_O_2_ and $\text{N}\,{\text{O}}_{2}^{-}$ together determine inactivation

**FIGURE 10:**
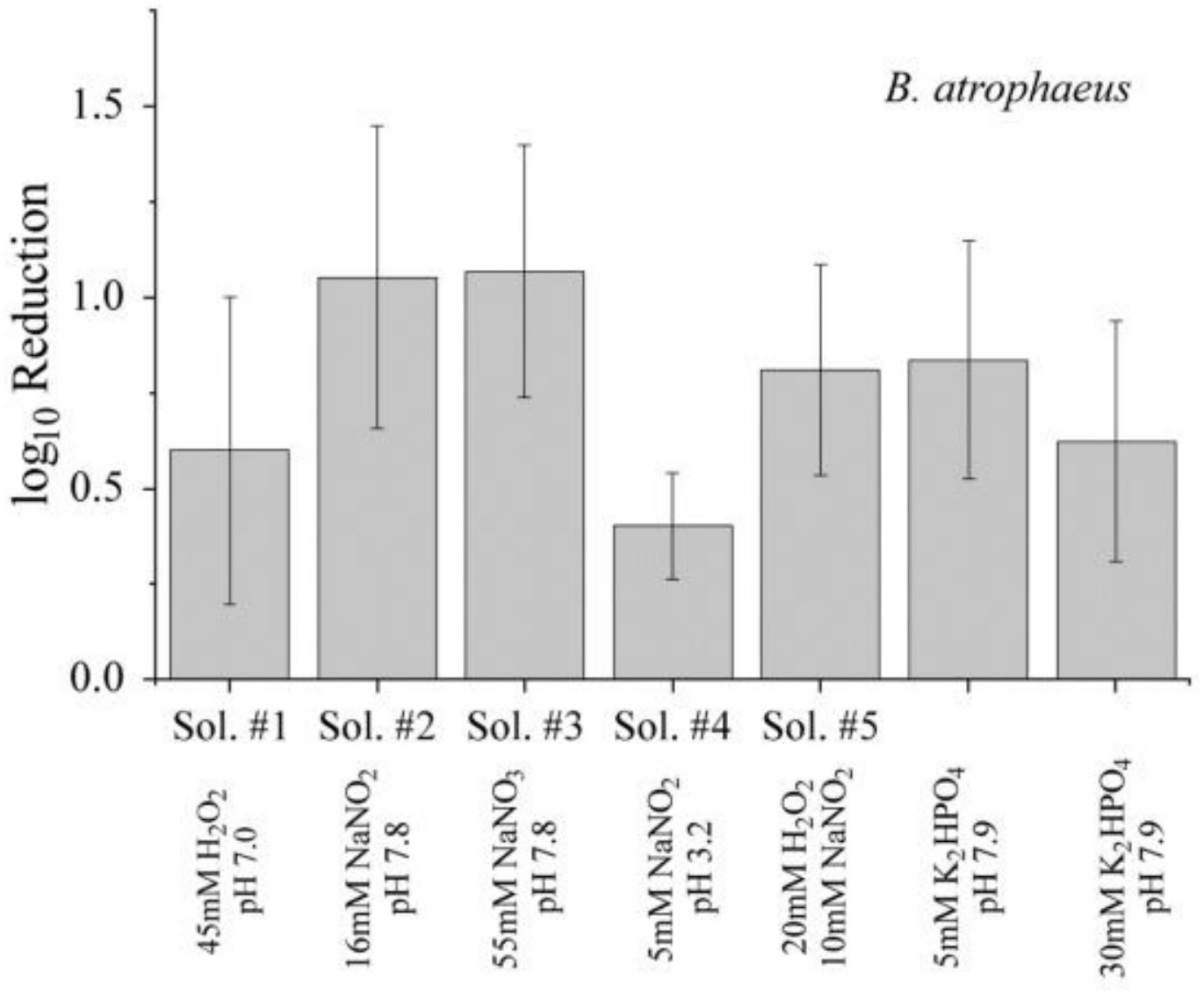
Inactivation of dormant *B. atrophaeus* spores (1–2⋅10^7^ cfu/mL) in aqueous solutions with defined H_2_O_2_ and NO_2_^–^ concentrations. Solutions were kept at room temperature and dormant *B. atrophaeus* spores were suspended immediately after the solutions were prepared. Bacteria were added 90 min after the solutions with H_2_O_2_ and NO_2_^–^ were mixed together to mimic the “post-reaction window.” As control, the bacteria were added to a solution with only 200 mM H_2_O_2_. Data correspond to exposure conditions and log_10_ reductions that are summarized for the different solutions in Tables 1, 2.

Microbial inactivation caused by hydrogen peroxide or nitrite alone was limited to less than one order of magnitude for concentrations of these species that were achieved by plasma treatment of 30 mM K_2_HPO_4_ buffer. However, together they could foster much better results. This was confirmed for solutions prepared with an excess of H_2_O_2_ in concentrations of 30 (Sol. #6), 40 (Sol. #7), 60 (Sol. #8), 100 (Sol. #9), 200 mM (Sol. #10; section 2.4.3). Concurrently, concentrations of $\text{N}\,{\text{O}}_{2}^{-}$ were adjusted to half the value of the respective hydrogen peroxide addition. Exact formulations can be found in Table 2. Solutions were kept at room temperature and *B. atrophaeus* spores were suspended immediately after the solutions were prepared (subsection 2.4.3). The results are shown in Figure 10. Even at 30 mM H_2_O_2_ with 15 mM $\text{N}\,{\text{O}}_{2}^{-}$, inactivation exceeded that previously observed for direct mechanisms. At 60 mM H_2_O_2_ and 30 mM of $\text{N}\,{\text{O}}_{2}^{-}$, the reduction reached more than four orders of magnitude. Potentially higher reductions at greater concentrations could not be quantified with the implemented procedure.

Moreover, a “post-reaction” experiment provided convincing evidence that the reaction of both components to form peroxynitrous acid was decisive for the observed inactivation of four orders of magnitude (Figure 10). When bacteria were added 90 min after a solution of 200 mM H_2_O_2_ and 100 mM $\text{N}\,{\text{O}}_{2}^{-}$ was prepared, inactivation was reduced to less than one order of magnitude. This indicated that any peroxynitrous acid responsible for the observed inactivation of four orders of magnitude, had likely already decomposed when the microorganisms were added. The critical role of combining hydrogen peroxide and nitrite was confirmed by suspending bacteria in a solution containing 200 mM H_2_O_2_ without added nitrite (Sol. #11). In this case, only a 1.5 log_10_ reduction was achieved, considerably lower than that obtained for the combination with $\text{N}\,{\text{O}}_{2}^{-}$. The experiments strongly support the conclusion that ONOOH formation is responsible for the observed inactivation within the exposure windows identified for plasma treatment of K_2_HPO_4_ solutions.

## 4 Discussion

**FIGURE 11:**
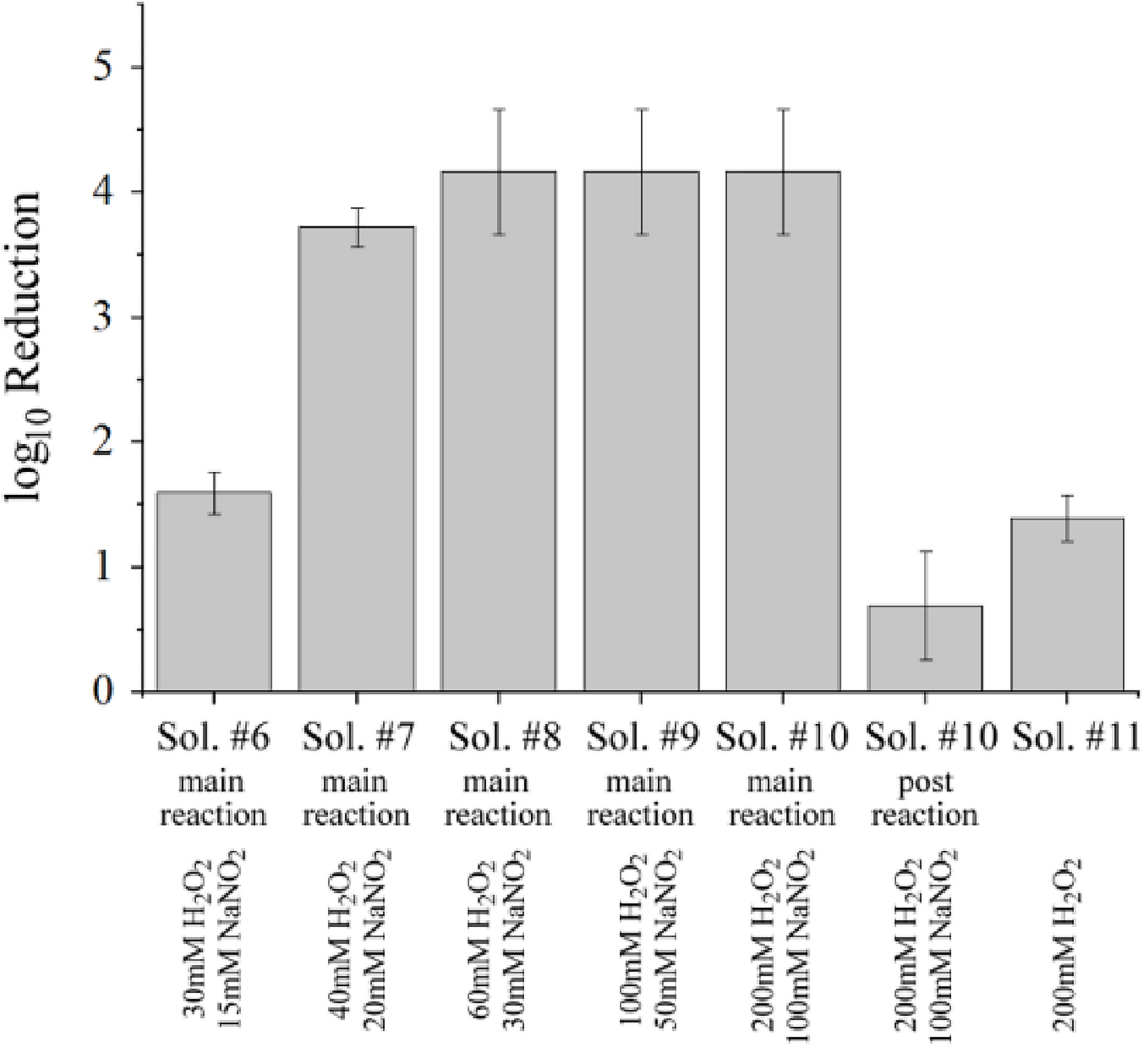
Inactivation of *E. coli* (vegetative cells) and *B. atrophaeus* (dormant spores) in a buffered solution (K_2_HPO_4_ buffer) or tap water in a concentration of 1–2⋅10^7^ cfu/mL that were directly treated by plasma for 3 min.

The antimicrobial potential of plasma-treated aqueous solutions has been repeatedly reported (Fan et al., 2024; Naitali et al., 2010; Oehmigen et al., 2011). While mechanisms of action such as elevated temperatures or UV emission may contribute, reactive species introduced into the liquid or generated by induced reaction chemistry are primarily responsible for the observed efficacy. Accordingly, only insignificant inactivation of *E. coli* or *B. atrophaeus* spores was achieved during short direct plasma exposures, which did not allow reactive plasma chemistry to develop (Figure 11) or merely caused a temperature increase (Figure 9).

During plasma treatment, the bulk liquid temperature was continuously monitored and did not exceed 32 °C even for the longest treatment durations (section 2.1). Nevertheless, to assess potential thermal contributions to microbial inactivation, dedicated control experiments were conducted in which plasma-treated buffer solutions as well as chemically prepared precursor systems (Sol. #1–#5) were equilibrated at 40 °C prior to microbial exposure; under these conditions, inactivation efficiencies were comparable to those obtained at room temperature (section 3.2.3) (Figures 8, 9).

Several studies have reported that elevating the solution temperature can enhance the antimicrobial efficacy of plasma-activated water, either when temperature is applied as an additional hurdle or when low-to-moderate temperatures are compared. For example, Xiang et al. showed that PAW alone resulted in only limited inactivation of *E. coli* O157:H7 (≈0.7 log_10_), whereas combining PAW with mild heat led to pronounced synergistic effects, achieving complete inactivation at 60 °C within a few minutes (Xiang et al., 2019). Similarly, Choi et al. reported improved microbial reduction when PAW treatment was sequentially combined with mild heating (50–60 °C) in food-related matrices, while PAW applied at ambient temperature yielded comparatively modest reductions (Choi et al., 2019). More recently, Fina et al. systematically analyzed such temperature effects and attributed the observed antimicrobial synergy to accelerated peroxynitrous acid formation kinetics from H_2_O_2_ and NO_2_^−^ under temperature-modulated conditions (Fina et al., 2024). Their results indicate that elevated temperatures increase reaction rates between precursor species, thereby shortening the time required to reach microbicidal ONOOH concentrations, consistent with Arrhenius-type kinetic acceleration.

In addition, hydrogen peroxide is known to exhibit temperature-dependent stability and reaction kinetics. Increasing temperature may accelerate both its formation and decomposition, thereby potentially altering downstream ONOOH generation dynamics (Goldstein et al., 2005; Vione et al., 2003). In the present work, however, no steeper accumulation or accelerated decay of H_2_O_2_ was observed in temperature-monitored measurements. Importantly, moderate temperature elevation did not measurably alter the concentration profiles or temporal evolution of H_2_O_2_ and NO_2_^−^ in plasma-treated solutions, indicating that precursor availability and subsequent ONOOH formation kinetics remained unaffected within the investigated temperature range.

Taken together, these results suggest that, under the present experimental conditions, antimicrobial activity is governed primarily by chemically defined ONOOH reaction windows rather than by temperature-driven synergistic effects. The apparent discrepancy to literature reports can thus be rationalized by differing experimental regimes: while elevated temperatures (≥ 50–60 °C) may act as an additional stressor and kinetic accelerator in other studies, temperature variations between room temperature and 40 °C do not significantly influence plasma-induced ONOOH chemistry in the present system.

Plasma-generated species include traditional disinfecting agents such as ozone, nitrogen oxides, and hydrogen peroxide. Nevertheless, these compounds alone cannot account for the high inactivation rates frequently observed, nor can selective admixtures of these species at concentrations measured under plasma treatment conditions reproduce those effects. This was specifically confirmed for solutions containing H_2_O_2_ and $\text{N}\,{\text{O}}_{2}^{-}$ individually, which are species found in plasma treatment (Figure 8). The contribution of ozone to the observed antimicrobial effects can be excluded, as its accumulation was effectively prevented by continuous air circulation using a fan during plasma operation.

Against this background, hydrogen peroxide (H_2_O_2_) and nitrite (NO_2_^−^) represent the central precursors for the formation of peroxynitrous acid (ONOOH), which has been discovered to be essential for achieving high microbial inactivation through plasma treatment. This is most prominently expressed by the impact on dormant spores, which are very resilient to traditional disinfecting agents. Accordingly, a 4-log_10_ reduction was consistently achieved when ONOOH formation was promoted by mixtures of H_2_O_2_ and NaNO_2_, as demonstrated in Figure 10.

The strong dependence of reported inactivation rates for plasma-treated solutions on experimental setup reflects both the transient behavior of ONOOH in aqueous environments and an incomplete understanding of the conditions needed to produce and sustain high concentrations over time. Therefore, the buffer capacity of the solution plays a crucial role in regulating pH and correspondingly ensures the accumulation of reactive species that are necessary for ONOOH synthesis. The motivation for the presented study was a detailed understanding of the reaction kinetics of ONOOH formation and decay with respect to the buffer capacity of an aqueous solution and for tap water as it is the most relevant liquid for many applications. Quantifying absolute concentrations and their temporal development has, for the first time, provided a detailed understanding of the conditions necessary to achieve maximal inactivation of microorganisms and dormant spores.

### 4.1 Critical assessment of reported antimicrobial efficacies of plasma-treated solutions

One of the first studies on the role of peroxynitrous acid for the inactivation of microorganisms in plasma-treated liquids was presented by Oehmigen et al. who found a ≥ 5 log_10_ reduction for *E. coli* in a non-buffered solution within 15 min of plasma exposure (Oehmigen et al., 2010). When a 10 mM phosphate-buffered solution (PBS) was used, microbial inactivation was significantly reduced, even after an extended treatment of 30 min; this reduction was attributed to the lack of acidification, which prevented the formation of reactive nitrogen species.

Our findings confirm that progressive acidification corresponds to an increased production of ONOOH. However, we challenge the notion that a decrease in pH is actually a prerequisite for microbial inactivation and find that it is rather the result of the underlying reaction chemistry. The buffered solution should initially allow for the accumulation of H_2_O_2_ and $\text{N}\,{\text{O}}_{2}^{-}$ until the buffer capacity is eventually exhausted and conversion rates to ONOOH increase with the associated acidification. Conversely, the 30 min treatment used by Oehmigen et al. may have simply been too short to deplete the buffer capacity of the PBS. In our experiments, buffer depletion required about 49 min, even for tap water. The presence of NaCl in PBS may also contribute to a lower availability of free $\text{N}\,{\text{O}}_{2}^{-}$ for ONOOH formation, further diminishing the antimicrobial potential of plasma-treated solutions (Fan et al., 2024).

While Oehmigen et al. used a dielectric barrier discharge for the generation of plasma-treated water, Naitali et al. examined the antimicrobial potential of water treated by a gliding arc discharge, focusing on long-lived reactive species like$\text{N}\,{\text{O}}_{2}^{-}$, $\text{N}\,{\text{O}}_{3}^{-}$, and H_2_O_2_ (Naitali et al., 2010). For *Hafnia alevi*, a gram-negative member of the *Enterobacteriaceae* family like *E. coli*, they obtained results comparable with Oehmigen et al. for *E. coli*. A reduction of > 5 log_10_ was achieved within 30 min. When solutions were buffered to maintain a pH value of 6 (with Na_2_HPO_4_ or KH_2_PO_4_), inactivation was minimal (<1 log_10_ reduction). Again, our results suggest that the high buffer capacity apparently prevented the reaction chemistry that produces ONOOH from being established, or would have required much longer treatment times to exhaust the buffer capacity.

Although both studies already emphasize the role of peroxynitrous acid for microbial inactivation, they also conclude that the associated acidification is crucial. The correlation was investigated in more detail by Lukeš et al. (2014), who demonstrated that rapid acidification actually enhanced the antimicrobial efficacy associated with increasing concentrations of ONOOH. For *E. coli*, a 3 log_10_ reduction was obtained after 30 min of the exposure of water to a plasma jet, with ONOOH concentrations reaching about 30 μM and pH dropping to between 2 and 3. However, since no buffers were added and only the inactivation of *E. coli* was pursued, no further details on inactivation and reaction dynamics, such as sustained high ONOOH concentrations, could be provided. Conversely, our results indicate that *E. coli* can be inactivated under highly acidic conditions alone, suggesting that its sensitivity may result from other factors, such as the combined effects of residual H_2_O_2_ and low pH, rather than ONOOH. In contrast, *B. atrophaeus* spores require the sustained presence of ONOOH for effective inactivation, as shown by the respective control experiments.

Acidification associated with peroxynitrous acid production was also the topic of a study by Heaselgrave et al. (2010) The combination of H_2_O_2_ and acidified NaNO_2_ led to a ≥ 4–log_10_ reduction for all tested microorganisms within 1 h, including highly resilient *Acanthamoeba* cysts and *Bacillus subtilis* spores. The essential role of peroxynitrous acid was reasoned from the only marginal inactivation ( ≤ 1 log_10_) of *Bacillus subtilis* spores, *Fusarium solani* conidia, and *Acanthamoeba* cysts by H_2_O_2_ or NaNO_2_ alone, even after immersion for 6 h.

Balazinski et al. (2021) investigated ONOOH-dependent inactivation in a chemically prepared system where ONOOH was generated *in situ* by mixing acidified H_2_O_2_ and NaNO_2_. Similar to our own suspension experiments with pre-mixed reactants (Figure 10), their results confirmed that ONOOH formation, rather than low pH or either reactant alone, is essential for effective microbial inactivation. Specifically, equimolar H_2_O_2_/NO_2_^–^ concentrations ≥ 10 mM at pH 3–4 produced ≥ 4 log_10_ reductions of *E. coli*; antimicrobial efficacy strongly depended on both pH and initial reactant concentrations.

In contrast, in plasma-treated solutions H_2_O_2_ and NO_2_^–^ are continuously generated in a non-stoichiometric ratio ([H_2_O_2_] ≳ 2 × [NO_2_^–^]), making NO_2_^–^ the limiting reactant during the main reaction phase and thus controlling ONOOH availability over time. We quantitatively confirmed Balazinski et al.’s qualitative observation on the decisive role of ONOOH by linking ONOOH formation dynamics in plasma-treated, buffered systems to inactivation windows.

### 4.2 Correlation of ONOOH reaction kinetics and antimicrobial efficacy

To explain microbial inactivation through the formation of peroxynitrous acid, three distinct phases were found crucial in the formation and decay of ONOOH (as shown in Figures 3–5). Reactive species were introduced into the liquid during plasma exposure, and in particular H_2_O_2_ and $\text{N}\,{\text{O}}_{2}^{-}$ accumulated in phase 1 (pre-reaction). This initial phase laid the foundation for ONOOH formation but did not yet demonstrate significant antimicrobial effects (Figures 6, 7). Eventually, H_2_O_2_ and $\text{N}\,{\text{O}}_{2}^{-}$ react to form ONOOH with turnover rates steadily increasing. This leads to peak concentrations of H_2_O_2_ and $\text{N}\,{\text{O}}_{2}^{-}$, subsequently, of ONOOH in phase 2 (main reaction). This phase was identified as the most critical for microbial inactivation (Figures 6, 7). Eventually the reaction kinetics shift toward the decomposition of ONOOH, as indicated by decreasing ONOOH concentrations and correspondingly increasing concentrations of HNO_2_ and $\text{N}\,{\text{O}}_{3}^{-}$, alongside residual concentrations of H_2_O_2_ and $\text{N}\,{\text{O}}_{2}^{-}$ in phase 3 (post-reaction). Inactivation of bacteria or spores added during this phase was minimal, demonstrating that acidification alone is insufficient without the presence of ONOOH (Figures 6, 7). Consequently, the three phases are associated with distinct pH changes and corresponding differences in absolute concentrations of reactive species. The time course of these changes strongly depends on the buffer capacity of the solution.

A comparison of 5 mM and 30 mM K_2_HPO_4_ buffers and tap water demonstrated that higher buffer capacity prolongs the availability of reactive species, delays acidification, and sustains ONOOH formation. Tap water exhibited a rapid drop in pH similar to the 5 mM buffer, which limited ONOOH stability to a shorter time window than that observed for the 30 mM buffer.

This assessment, can be confirmed by simulations. In a buffer with 5 mM K_2_HPO_4_, a maximum ONOOH concentration of approximately 38 μM was reached with levels above 20 μM maintained for about 7 min. For 30 mM K_2_HPO_4_, simulated peak concentrations approached 110 μM and remained above 50 μM for more than 10 min. Notably, the development of reaction precursors and degradation compounds, including H_2_O_2_, $\text{N}\,{\text{O}}_{2}^{-}$, HNO_2_, and $\text{N}\,{\text{O}}_{3}^{-}$, as well as changes in pH are consistent with the measurements. The higher buffer capacity at 30 mM stabilized the pH for a longer time, facilitated sustained accumulation of H_2_O_2_ and $\text{N}\,{\text{O}}_{2}^{-}$, and thereby prolonged ONOOH availability.

Upon depletion of $\text{N}\,{\text{O}}_{2}^{-}$, ONOOH generation effectively ceased, and the reaction kinetics shifted toward a post-reactive regime characterized by ONOOH decay and accumulation of stable products such as $\text{N}\,{\text{O}}_{3}^{-}$. Residual H_2_O_2_, persisting in measurable concentrations, likely maintained mild oxidative conditions that continued to affect vegetative bacterial cells, as explained previously (Otter and French, 2009; Sakudo et al., 2017), whereas spores were largely unaffected during this phase. The corresponding oxidative environment may have contributed to the overall antimicrobial efficacy observed for *E. coli*, even after the more effective inactivation associated with peak values of ONOOH concentrations became collateral.

Overall, the simulations elucidate the coupled production and decay kinetics underlying peroxynitrous acid dynamics. This mechanistic understanding provides a quantitative framework for optimizing plasma treatment parameters, such as buffer strength, reactant supply, and exposure duration, to maximize antimicrobial efficacy. It also lays the foundation for transferring plasma treatment strategies from controlled laboratory systems to real-world scenarios with variable buffering capacity, pH, and microbial resistance.

### 4.3 Control of inactivation windows based on ONOOH reaction kinetics

Reaction kinetics of ONOOH and the associated possibility of a control provides a “time window” in which the inactivation of vegetative bacteria and resilient spores can be maximized. This “maximum window” is defined by the transient stability of ONOOH before it decomposes into less reactive stable species (Figures 3–5). The underlying reaction chemistry of HNO_2_ and H_2_O_2_ was described by Vione et al., demonstrating the formation of ONOOH (Equations 2–6) (Vione et al., 2003). Formation of ONOOH was found to be pH dependent with the reaction being more efficient under acidic conditions. Eventually, ONOOH decomposes into OH⋅ and NO_2_.

In contrast to classical chemical systems with fixed initial reactant concentrations (such as HNO_2_ and H_2_O_2_), reactive precursors are continuously supplied during plasma treatment, resulting in dynamic concentration changes and progressive acidification. Accordingly, buffer systems can modulate both the temporal and quantitative profiles of ONOOH formation and decay, providing a means to control the associated antimicrobial inactivation.

Plasma-treated aqueous solutions exert antimicrobial effects through multiple pathways, including oxidative stress, membrane disruption, and DNA or protein oxidation (Kashket, 1987; Shen et al., 2019). While decreasing pH generally intensify these effects, acidification alone is not the primary driver. This is evident from the inactivation patterns observed for different buffer systems. *E. coli* was effectively inactivated in both buffered and non-buffered media, with log_10_ reductions exceeding 5 orders for a solution with 5 mM K_2_HPO_4_ and of 3.84 orders for tap water when cells were added during phase 2 at peak ONOOH concentrations. In contrast, addition of the cells during phase 3, when ONOOH levels had declined, resulted in markedly weaker effects despite a low pH.

For *B. atrophaeus* spores, efficient inactivation required the coincidence of both acidification and high ONOOH availability. In a 5 mM K_2_HPO_4_ solution, spore reduction remained < 1 log_10_ at a pH of 2.73 (phase 3), whereas in a 30 mM buffer with a pH of 4.01 (phase 2), a reduction > 3 log_10_ was achieved. Tap water behaved similarly to the weaker buffer ( ≤ 0.41 log_10_ for spores, 3.84 log_10_ for vegetative bacteria), confirming that limited buffering permits sufficient ONOOH formation for vegetative cells but not for spores. Hence, buffer capacity emerges as a key control parameter: higher phosphate concentrations sustain ONOOH accumulation and extend its lifetime, which markedly enhances antimicrobial efficacy.

The transient nature of ONOOH was corroborated by different defined experiments. Freshly mixed solutions with H_2_O_2_ (30 mM) and $\text{N}\,{\text{O}}_{2}^{-}$ (15 mM) resulted in a > 4 log_10_ reduction of spores, whereas pre-incubation for 90 min before exposure reduced efficacy to < 1 log_10_. Solutions containing exclusively either H_2_O_2_ or $\text{N}\,{\text{O}}_{2}^{-}$ were ineffective (< 1 log_10_ at a pH of 3.2). These findings confirm that ONOOH must be generated *in situ* and be present at the time of microbial contact to exert strong antimicrobial activity. During plasma exposure, progressive acidification of the solutions was observed, concurrent with distinct reaction kinetics across media. The duration of phase 1—accumulation of reactive species to peak values—correlated with the time required to deplete buffering capacity (276 min for 30 mM, 52 min for 5 mM, and 49 min for tap water). Excessive buffering that cannot be exhausted during plasma treatment limits ONOOH formation and thus microbial inactivation. This is consistent with, and explains, the weak inactivation that was reported by Oehmigen and Heaselgrave in highly buffered systems (Heaselgrave et al., 2010; Oehmigen et al., 2010).

In summary, optimized buffering enhances ONOOH accumulation and prolongs its effective lifetime, enabling robust inactivation of both *E. coli* cells and *B. atrophaeus* spores. Maintaining sufficient concentrations of H_2_O_2_ and $\text{N}\,{\text{O}}_{2}^{-}$ during plasma exposure is critical to sustain ONOOH formation. Tailoring plasma parameters, treatment duration, temperature, and exposure intensity to the buffering characteristics of the treated medium is therefore essential for maximizing antimicrobial efficacy in practical applications.

## 5 Conclusion

This study provides a detailed understanding of the reaction chemistry responsible for the inactivation of vegetative bacterial cells and dormant spores by the formation of peroxynitrous acid during plasma treatment of aqueous solutions. High and sustained concentrations of ONOOH are particularly important to achieve high reductions of sporicidal forms. In this case, other reactive species or mechanisms, such as mere acidification, are much less effective despite relatively low absolute concentrations of ONOOH. Therefore, treatments and solutions have to be devised to guarantee a sufficient and sustained accumulation of ONOOH. This can be controlled by the buffer capacity of the solution. Higher buffer concentrations result in extended production times. However, they should not be so high that the buffer capacity is not eventually exhausted during plasma treatment, because depletion allows for accumulated $\text{N}\,{\text{O}}_{2}^{-}$ and H_2_O_2_ to convert to ONOOH and reach respectively high concentrations. Additionally, three distinct phases can be identified for the crucial evolution of reactive species: (1) the introduction and accumulation of reactive species; (2) increasing conversion to ONOOH after the buffer capacity is exhausted; and (3) a shift to reaction kinetics that favor ONOOH decomposition as the solution becomes increasingly acidified. An extended second phase is particularly important for efficient inactivation.

In the present study, peroxynitrous acid cannot be quantified directly due to its short lifetime and low steady-state concentrations. Instead, the formation and decay of the acid were inferred from precursor species kinetics supported by numerical simulations and chemically analogous inactivation systems. While this indirect approach introduces a degree of uncertainty, the convergence of spectroscopic data, kinetic modeling, and microbiological outcomes provides consistent mechanistic evidence for ONOOH-driven inactivation.

Furthermore, experiments were conducted in defined phosphate buffer systems to enable controlled modulation of buffer capacity and reaction kinetics. Although this approach allows mechanistic resolution, transferability to more complex aqueous or biological matrices may be influenced by additional constituents and buffering effects.

Finally, the treatment times required to reach optimal inactivation windows reflect the use of a low-power laboratory discharge specifically designed for mechanistic investigations rather than for process optimization. The present study therefore aims at resolving the underlying chemical pathways governing antimicrobial efficacy. For practical implementation, further development and optimization of plasma sources—such as increased power densities, improved energy coupling, or reactor scaling—will led to shorter treatment times higher treatment volumes. These steps will be required to translate the identified reaction kinetics into efficient treatment regimes.

The mechanistic insights gained in this work are likewise applicable to plasma treatment of water beyond defined buffer systems. Although the buffer capacity of tap water is inherently lower, it still promotes the same fundamental chemical evolution of plasma-induced reactive species. Based on this understanding, treatment conditions such as exposure times can be adapted accordingly. This provides a rational basis for improving disinfection strategies in wastewater treatment, food sanitation during washing processes, and the decontamination of organic materials in medical and bioengineering applications.

## Data Availability

The original contributions presented in this study are included in this article/supplementary material, further inquiries can be directed to the corresponding author.
